# Tdp1 processes chromate-induced single-strand DNA breaks that collapse replication forks

**DOI:** 10.1371/journal.pgen.1007595

**Published:** 2018-08-27

**Authors:** Abantika Ganguly, Lan Guo, Lingling Sun, Fang Suo, Li-Lin Du, Paul Russell

**Affiliations:** 1 Department of Molecular Medicine, The Scripps Research Institute, La Jolla, California, United States of America; 2 National Institute of Biological Sciences, Beijing, People’s Republic of China; National Institute of Environmental Health Sciences, UNITED STATES

## Abstract

Hexavalent chromium [Cr(VI)] damages DNA and causes cancer, but it is unclear which DNA damage responses (DDRs) most critically protect cells from chromate toxicity. Here, genome-wide quantitative functional profiling, DDR measurements and genetic interaction assays in *Schizosaccharomyces pombe* reveal a chromate toxicogenomic profile that closely resembles the cancer chemotherapeutic drug camptothecin (CPT), which traps Topoisomerase 1 (Top1)-DNA covalent complex (Top1cc) at the 3’ end of single-stand breaks (SSBs), resulting in replication fork collapse. ATR/Rad3-dependent checkpoints that detect stalled and collapsed replication forks are crucial in Cr(VI)-treated cells, as is Mus81-dependent sister chromatid recombination (SCR) that repairs single-ended double-strand breaks (seDSBs) at broken replication forks. Surprisingly, chromate resistance does not require base excision repair (BER) or interstrand crosslink (ICL) repair, nor does co-elimination of XPA-dependent nucleotide excision repair (NER) and Rad18-mediated post-replication repair (PRR) confer chromate sensitivity in fission yeast. However, co-elimination of Tdp1 tyrosyl-DNA phosphodiesterase and Rad16-Swi10 (XPF-ERCC1) NER endonuclease synergistically enhances chromate toxicity in *top1Δ* cells. Pnk1 polynucleotide kinase phosphatase (PNKP), which restores 3’-hydroxyl ends to SSBs processed by Tdp1, is also critical for chromate resistance. Loss of Tdp1 ameliorates *pnk1Δ* chromate sensitivity while enhancing the requirement for Mus81. Thus, Tdp1 and PNKP, which prevent neurodegeneration in humans, repair an important class of Cr-induced SSBs that collapse replication forks.

## Author summary

Hexavalent chromium is a carcinogen that is found at toxic waste sites and in some groundwater supplies. Cellular metabolism converts chromium into DNA-damaging chromate, but it is unclear which types of chromate-DNA lesions are most dangerous, and which cellular mechanisms most critically prevent chromium toxicity. This study uses whole-genome profiling to identify DNA repair pathways that are crucial for chromate resistance in fission yeast. The resulting ‘toxicogenomic’ profile of chromate closely matches camptothecin, a natural product representing a class of chemotherapeutic drugs that cause replication fork collapse by poisoning Topoisomerase 1 (Top1), which relaxes supercoiled DNA by creating and resealing single-strand breaks (SSBs). Genetic interaction analyses uncover important roles for Tdp1 tyrosyl-DNA phosphodiesterase and Pnk1 polynucleotide 5’-kinase 3’-phosphatase (PNKP), which repair camptothecin-induced SSBs and prevent neurological disease in humans. However, chromium toxicity does not involve Top1. As Tdp1 and Pnk1 repair SSBs with 3’-blocked termini, these data suggest that Top1-independent 3’-blocked SSBs contribute to the carcinogenic and mutagenic properties of chromium.

## Introduction

Chromium, with its unique combination of hardness, corrosion-resistance and lustrous appearance, is an exceptionally important industrial metal [1]. However, when handled improperly, hexavalent chromium [Cr(VI)] compounds pose serious risks to human health. Occupational exposures have provided compelling evidence that Cr(VI) is a respiratory carcinogen [2], and mouse studies established that Cr(VI) in drinking water causes tumors in the alimentary canal [3]. For these reasons, chronic exposure to Cr(VI) through groundwater contamination is a significant health concern [4]. However, there is no consensus on the molecular mechanisms by which chromium causes malignancy [5–8]. Cr(VI) does not interact with DNA, but intracellular reduction traps protein and DNA-reactive chromate products [Cr(V), Cr(IV) and Cr(III)] inside cells. These chromates may cause DNA damage through Cr-DNA adduct formation, DNA interstrand crosslinking or DNA-protein crosslinks (DPCs) [6, 9–11]. Free radicals generated during Cr(VI) reduction might also promote carcinogenesis through oxidative base modification and mutagenesis [11].

It is currently unclear which DNA damage responses (DDRs) most critically protect cells from chromate [10–12]. Virtually every DNA repair pathway has been implicated in chromate resistance, including nucleotide excision repair (NER), base excision repair (BER), mismatch repair (MMR), inter-strand crosslink (ICL) repair and translesion DNA synthesis (TLS), as well as double-strand break (DSB) repair by nonhomologous end-joining (NHEJ) or homologous recombination (HR). Recent studies have focused on interference of DNA replication by Cr-DNA adducts, which might cause replication fork collapse [13–16]. Replication of Cr-adducted DNA might also be required to allow MMR nucleases to create DSBs [17, 18]. Activation of ATM and ATR checkpoint kinases has also been reported in chromium-treated cells [19–22].

Whole genome deletome libraries of model organisms provide the means to systematically and quantitatively assess the importance of thousands of genes in determining toxicant sensitivity [23–27]. In *Saccharomyces cerevisiae*, two such unbiased screens identified 387 genes required for chromate resistance [28, 29]. DDR genes were in these lists but there was only marginal enrichment of DDR-associated gene ontology (GO) terms. Here, we combine deletome functional profiling, fitness assays, genetic interaction tests and DNA damage measurements to identify genes that are most critical for chromate resistance in the fission yeast *Schizosaccharomyces pombe*. DDR checkpoint genes are highly enriched in this screen, and we unexpectedly uncover important roles for tyrosyl-DNA phosphodiesterase and polynucleotide kinase phosphatase, which repair the 3’-blocked ends of single strand DNA breaks (SSBs). These proteins prevent neurodegenerative diseases in humans [30].

## Results

### Rad3/ATR-dependent DNA replication stress and DNA damage checkpoints are crucial for chromium resistance

Parallel mutant phenotyping by barcode sequencing (Bar-seq) [26, 27] was used to identify haploid deletion mutants that are sensitive to 10 μM potassium dichromate (K_2_Cr_2_O_7_), which slightly inhibits the growth of wild type (described in Materials and Methods). Growth inhibition (GI) scores were obtained for 2,887 mutants, leading to the identification of 68 genes required for chromate resistance (S1 Table). Most of these genes have orthologs in *S*. *cerevisiae* (59/68) or humans (52/68) (S1 Table), although of the *S*. *cerevisiae* orthologs, only 9 are amongst the 387 genes found in functional profiling screens performed with 100 or 900 μM CrO_3_ [28, 29].

**Fig 1:**
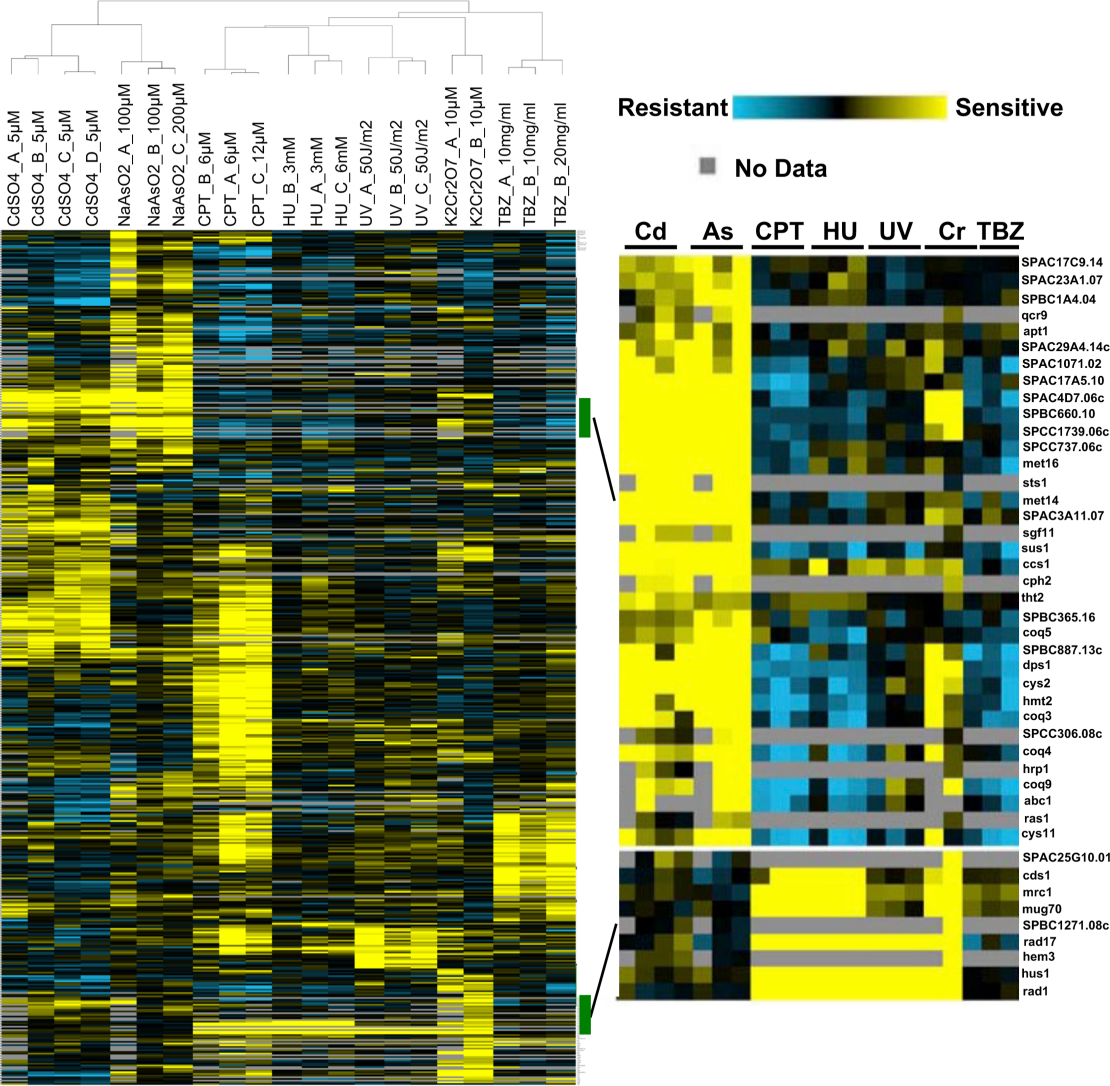
Hierarchical clustering of toxin-sensitive mutants. Mutants identified as toxin-sensitive in at least one functional profiling screen were subjected to two-dimensional hierarchical clustering analysis using their GI scores. Toxin concentrations for each screen are indicated at the top of each column. GI values were obtained from the chromium screens reported in this study or published screens using the same functional profiling platform [26, 27]. Zoom view (right panel, upper half) indicates mutants shared in cadmium and arsenic screens but not chromium. Right panel, lower half indicates Cr(VI)-sensitive mutants shared with genotoxin-sensitive screens (CPT, HU and UV) but not cadmium or arsenic. Thiabendazole (TBZ) is a microtubule-depolymerizing drug.

The web-based tool AnGeLi [31] identified “DNA replication checkpoint” and “DNA damage checkpoint” as two of the most highly enriched GO Biological Functional Categories (Table 1). Sensitivity to DNA damaging agents, such as camptothecin (CPT), hydroxyurea (HU), ionizing radiation (IR), and ultraviolet (UV) light, were amongst the most highly enriched phenotypes annotated to these genes (Table 2). Indeed, hierarchical cluster analysis revealed that the chromium profile more closely resembles DNA damaging agents (CPT, HU and UV) than the toxic metal cadmium or metalloid arsenic (Fig 1) [26, 27].

**Table 1:** GO Biological processes enriched for Cr(VI)-sensitive mutants.

GO Biological Process	Corrected p-value	Cluster Frequency (68 genes)	Genome Frequency (2901 genes)	Genes Annotated
DNA Replication checkpoint	2.69e-10	8 (11.8%)	9 (0.3%)	crb2, mrc1, hus1, cds1, rad1, rad9, ctp1, rad17
DNA damage checkpoint	1.01e-09	10 (14.7%)	19 (0.7%)	mrc1, rad26, rad1, rad9, ctp1, rad17, crb2, hus1, rad24, cds1
Chemical Homeostasis	0.0027	9 (13.2%)	58 (2.0%)	ptn1, vph2, rav1, cgs2, hmt2, kha1, fep1, pka1, cuf1

GO term enrichment performed against the *S*. *pombe* haploid deletion collection of 2901 genes, with Bonferroni correction, P-value cutoff = 0.01, FDR = 0% for all data in table, 0 false positives.

**Table 2:** Summary of annotated phenotypes enriched for Cr(VI)-sensitive mutants.

Phenotypes (FYPO)	p- value	List Frequency/ 68 genes	Background Frequency/ 5136 genes*	Genes
Sensitive to Camptothecin	1.07e-17	29 (42.65%)	248 (4.83%)	*SPBC16H5*.*13*, *SPCC1442*.*04c*, *apl5*, *apl6*, *cgs2*, *crb2*, *csn1*, *ctp1*, *ddb1*, *est1*, *fep1*, *hus1*, *mrc1*, *mug70*, *ngg1*, *pka1*, *rad1 rad17*, *rad24*, *rad26*, *rad9 rav1*, *rtt109*, *sgf73*, *tcg1*, *trt1*, *ubp8*, *ubp9*, *uge1*
Sensitive to Cisplatin	5.76e-12	18 (26.47%)	106 (2.06%)	*SPAC17H9*.*08*, *SPBC16H5*.*13*, *apl6*, *arp8*, *coq3*, *csn1*, *ctp1*, *dps1*, *est1*, *hap2*, *hus1*, *ngg1*, *nht1*, *rad1*, *rad17*, *rad24*, *rad9*
Sensitive to Hydroxyurea	9.50e-12	31 (45.59%)	580 (11.29%)	*SPBC16H5*.*13*, *apl5*, *apl6*, *cds1*, *crb2*, *csn1*, *ctp1*, *cys2*, *ddb1*, *dps1*, *est1*, *fep1*, *hus1*, *mrc1*, *mug70*, *ngg1*, *nht1*, *rad1*, *rad17 rad24*, *rad26*, *rad9*, *rav1*, *rtt109*, *sgf73*, *tcg1*, *ubp8*, *ubp9*, *vph2*, *vps20*, *vps28*
Sensitive to Cadmium	5.25e-06	18 (26.47%)	249 (4.85%)	*arp8*, *coq3*, *csx1*, *cuf1*, *cys11*, *ent3*, *hap2*, *hmt2*, *met8*, *ngg1*, *pcr1*, *rav1*, *rav2*, *sgf73*, *vsl1*
Sensitive to Ionizing Radiation during vegetative growth	5.43e-06	11 (16.18%)	73 (1.42%)	*crb2*, *ctp1*, *ddb1*, *hus1*, *met6*, *rad1*, *rad17*, *rad24*, *rad26*, *rad9*, *rtt109*
Sensitive to UV during vegetative growth	1.88e-04	13 (19.12%)	152 (2.96%)	*cds1*, *crb2*, *csn1*, *ctp1*, *ddb1*, *est1*, *hus1*, *rad1*, *rad17*, *rad24*, *rad26*, *rad9*, *trt1*

Screened against 5136 protein encoding genes, with Bonferroni correction, P-value cutoff 0.01.

**Fig 2:**
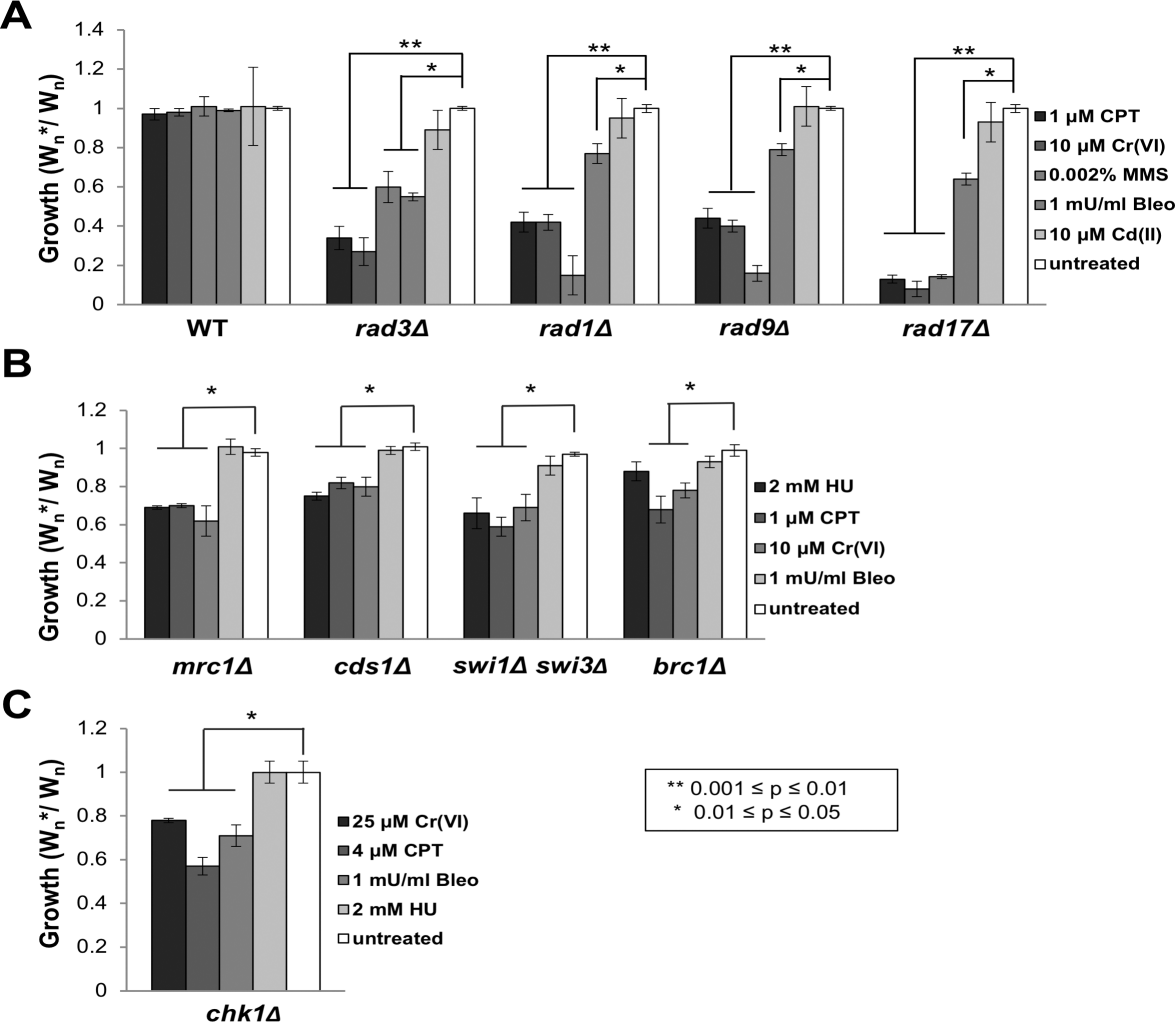
DNA replication stress and DNA damage checkpoint genes are required for chromium-6 resistance. Flow cytometry-based competitive growth assays determined mutant sensitivities to the indicated concentrations of toxicants. **(A)** Mutants lacking subunits of the Rad3/ATR checkpoint kinase, Rad9-Hus1-Rad1 checkpoint clamp or its Rad17-RFC clamp loader are sensitive to Cr(VI), CPT, MMS and bleomycin (Bleo), but not cadmium. **(B)** Mutants lacking the replication checkpoint kinase Cds1, the Mrc1 mediator required for Cds1 activation, the Swi1-Swi3 fork protection complex, or the multi-BRCT protein Brc1 are sensitive to Cr(VI), CPT, and HU (except *brc1Δ*), but not bleomycin. **(C)** A mutant lacking the DNA damage checkpoint kinase Chk1 is sensitive to Cr(VI), CPT and bleomycin, but not HU. The assays with *chk1Δ* cells used higher concentrations of Cr(VI) and CPT because *S*. *pombe* has a long G2 phase, hence *chk1Δ* cells are relatively less sensitive to genotoxins that collapse DNA replication forks. Statistically significant differences as determined by a two-tailed Student’s t-test are indicated. Bars represent standard deviation from three independent experiments.

As described in Materials and Methods, we adapted a flow cytometry–based growth competition assay to further examine Cr-sensitive mutants (S1 Fig) [32, 33]. This assay normalizes for slow growing mutants, which is essential to accurately assess many DDR mutants. Focusing on mutants that were absent from deletome library, we found that the master checkpoint kinase Rad3/ATR, which detects Replication Protein A (RPA) bound to ssDNA at stalled replication forks and resected DSBs [34], is crucial for chromate resistance (Fig 2A). For *rad3Δ* cells, the growth inhibition caused by 10μM chromate was similar to 1μM CPT. The functions of Rad3 largely depend on a PCNA-like DNA clamp consisting of Rad9, Hus1 and Rad1, and the corresponding clamp loader that includes Rad17 [35]. The growth competition assay confirmed that the ‘9-1-1’ checkpoint clamp and its loader are crucial for chromium resistance (Fig 2A). In response to replication fork arrest, Rad3 phosphorylates and activates Cds1/CHK2 kinase by a mechanism that requires Mrc1/CLASPIN and the Swi1-Swi3 fork protection complex, which is orthologous to human TIMELESS-TIPIN [35]. The growth competition assay confirmed that Cds1, Mrc1 and Swi1/Swi3 are important for Cr(VI) resistance (Fig 2B). Rad3 also phosphorylates the C-terminus of histone H2A in chromatin flanking stalled and damaged replication forks [36]. Phospho-H2A, known as γH2A in fission yeast, recruits Brc1 (PTIP/Rtt107 homolog) through its C-terminal pair of BRCT (BRCA1 C-terminal) domains [37]. We found that *brc1Δ* cells are sensitive to chromate (Fig 2B). Replication fork collapse creates seDSBs, which are detected by the γH2A-binding protein Crb2, which mediates phosphorylation of Chk1 checkpoint kinase by Rad3 [38]. The growth competition assay revealed that Chk1 is required for chromium resistance (Fig 2C), which is consistent with functional profiling data showing that *crb2Δ* cells are also Cr-sensitive.

### Cr-induced DNA lesions activate checkpoints in S-phase

**Fig 3:**
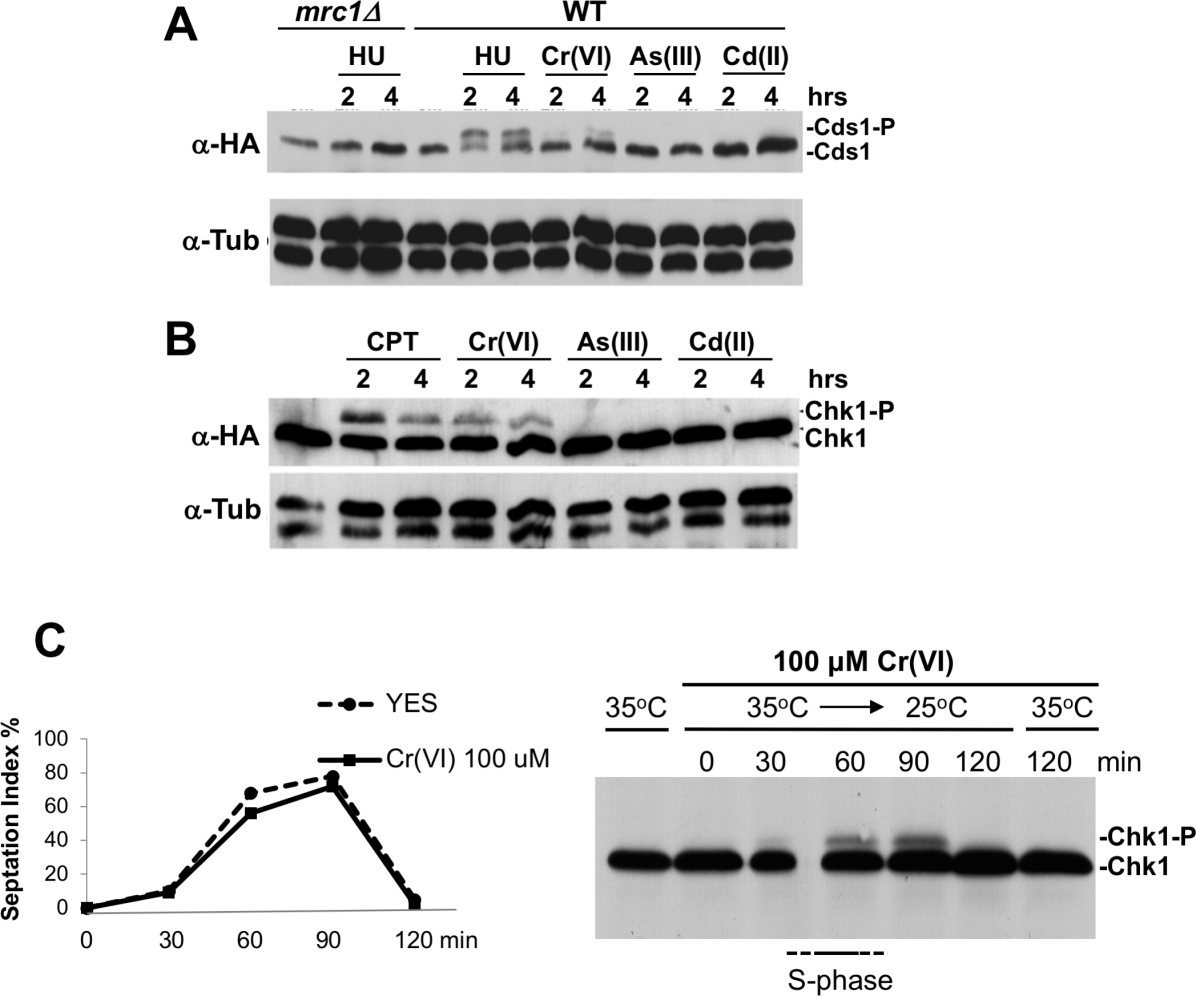
Hexavalent chromium activates Cds1 and Chk1 checkpoint kinases. **(A)** Exposure to Cr(VI), but not As(III) or Cd(II), triggers Cds1 phosphorylation, as detected by reduced mobility of HA-tagged Cds1 in SDS-PAGE. HU was used as a positive control. The absence of Cds1 phosphorylation in HU-treated *mrc1Δ* cells further validates the assay. Cells were incubated with toxicants for 2 or 4 hours at 30°C. Toxicant concentrations were 20 mM HU, 200 μM K_2_Cr_2_O_7_, 200 μM CdSO_4_ or 200 μM NaAsO_2_. Tubulin (anti-tub) was used as the loading control. **(B)** Exposure to Cr(VI), but not As(III) or Cd(II), triggers Chk1 phosphorylation, as detected by reduced mobility of HA-tagged Chk1 in SDS-PAGE. CPT was used as a positive control. Cells were incubated with toxicants for 2 or 4 hours at 30°C. Toxicant concentrations were 30 μM CPT, 200 μM K_2_Cr_2_O_7_, 200 μM CdSO_4_ or 200 μM NaAsO_2_. **(C)** Cr(VI)-treated cells activate Chk1 upon transit through S-phase. Temperature sensitive *cdc25-22* cells were synchronized in late G2 by incubation at 35°C for 3 hours, followed by an additional 2-hour incubation at 35°C in the presence of 100 μM Cr(VI). At time point 0, cells were released from the cell cycle block by lowering the temperature to 25°C. Activation of HA-tagged Chk1 was monitored by immunoblotting. Chk1 phosphorylation coincides with the rise in septation index (upper panel), which correlates with passage through S-phase. No Chk1 phosphorylation was observed in Cr(VI)-treated cells maintained in late G2 by incubation for an additional 120 minutes at 35°C (rightmost lane). The leftmost lane is the untreated control.

In agreement with the chromate sensitivity assays, immunoblotting confirmed that Cds1 and Chk1 undergo Rad3-dependent activating phosphorylation in response to chromium treatment (Fig 3A and 3B). Neither cadmium or arsenic had this effect, which is consistent with our previous functional profiling studies [26].

Cds1 checkpoint responses are specific for S-phase [39, 40], which suggested that the Chk1-activating lesions might also appear during replication. To address this question, we used the temperature sensitive *cdc25-22* mutation to arrest cells in G2 phase [41]. Chromate exposure during this arrest did not cause activating phosphorylation of Chk1 (Fig 3C, lane 2). However, upon reactivating Cdc25, Chk1 was phosphorylated as cells passed through S-phase (Fig 3C). Thus, collapse of replication forks at Cr-DNA lesions likely triggers Chk1 activation.

**Fig 4:**
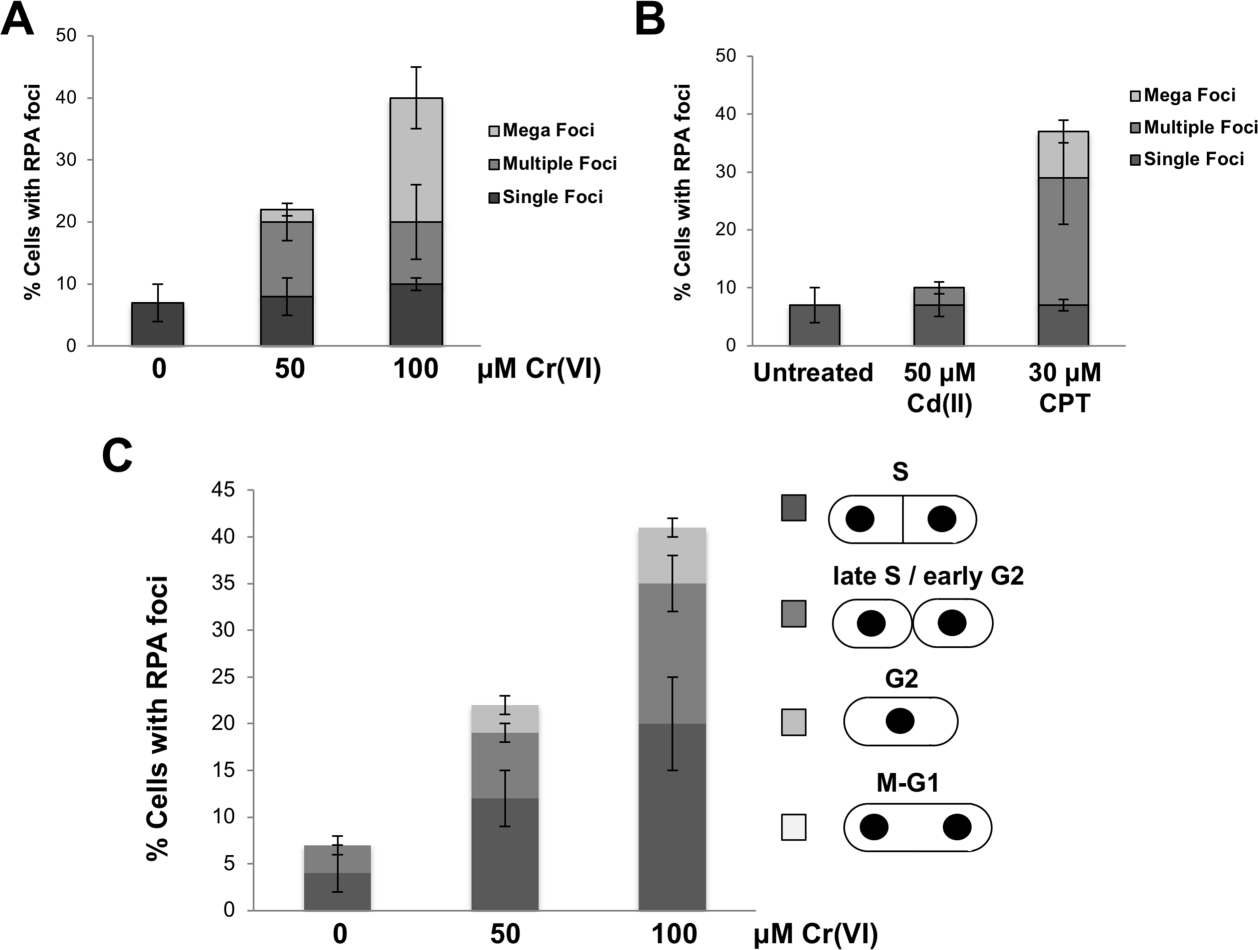
Increased RPA foci are observed following chromate exposure. **(A)** Increased number of RPA-GFP foci are detected in cells treated with 50 or 100 μM Cr(VI) for 2 hours at 25°C. Mega-foci, which are potentially clusters of RPA-GFP foci, are indicated by arrows. **(B)** Increased number of RPA-GFP foci are detected in cells treated with CPT. There was no change in RPA-GFP foci in cadmium-treated compared to untreated cells. Cells were treated with toxicants for 2 hours at 25°C. **(C)** Most Cr(VI)-treated cells with RPA-GFP foci are in S-phase or early G2 phase. Cell cycle stage was estimated from cell and nuclear morphology. Bars represent standard deviation from 3 independent experiments.

We also monitored RPA foci formation in live cells using green fluorescent protein (GFP)-tagged Ssb1/Rpa1. Chromate caused a large increase in RPA foci, with many cells containing multiple foci or large ‘mega-foci’ (Fig 4A). Similar responses were observed in CPT treated cells (Fig 4B). Analysis of cell cycle markers indicated that the majority of Cr-induced RPA foci appeared during S or early G2 phase (Fig 4C), as predicted from the Chk1 activation assays.

### Mus81-dependent SCR is critical in Cr-treated cells

**Fig 5:**
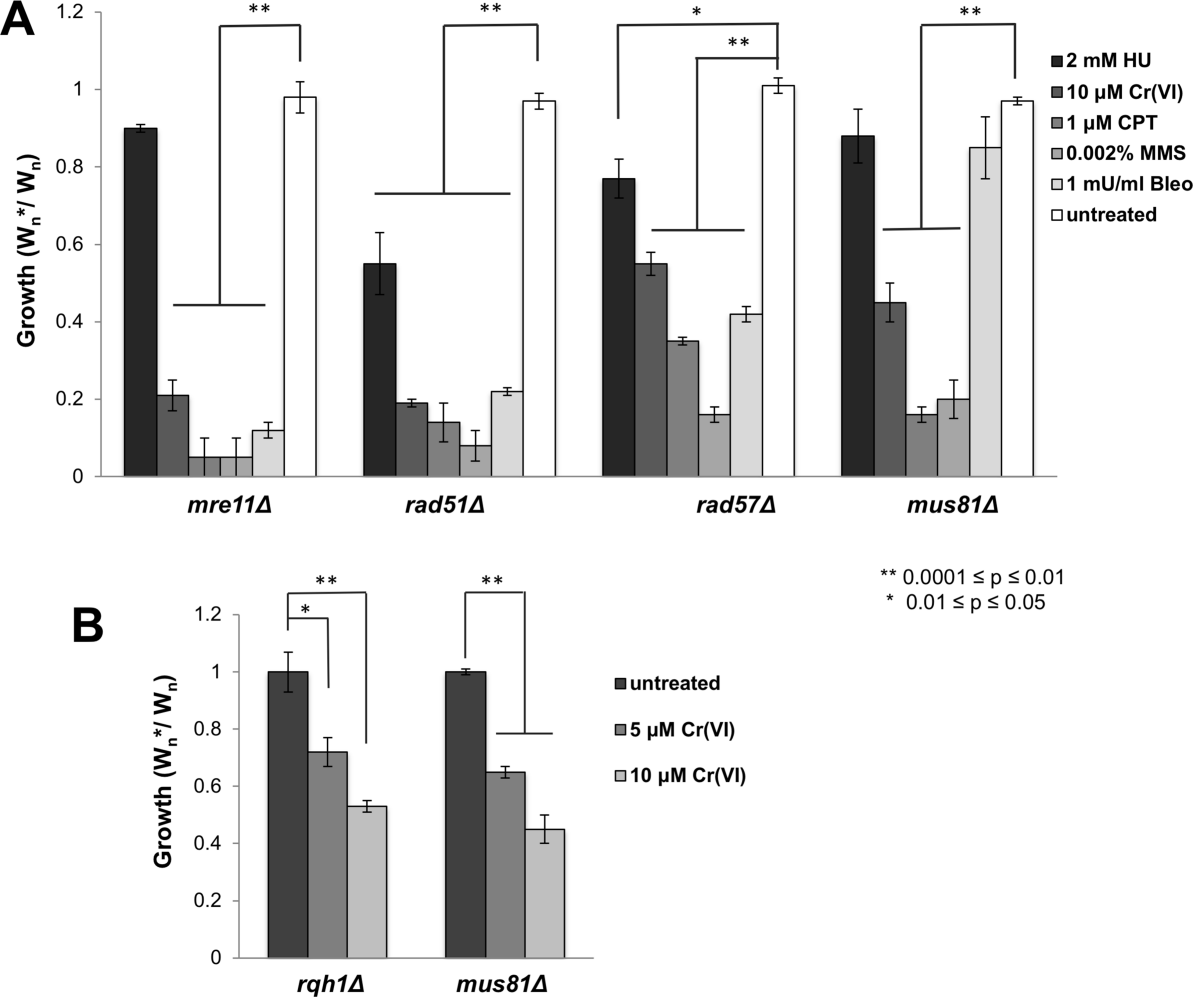
HR repair is crucial for chromium-6 resistance. **(A)** Flow cytometry-based competitive growth assays reveal that the Mre11 subunit of MRN DNA-end processing endonuclease, Rad51 and Rad57 recombinases, and Mus81 resolvase, are essential for resistance to Cr(VI). Note that all mutants were significantly sensitive to the indicated concentrations of Cr(VI), CPT and MMS, whereas only *rad51Δ* and *rad57Δ* were sensitive to HU and bleomycin. **(B)** RecQ DNA helicase is important for growth in the presence of Cr(VI). The sensitivity of *rqh1Δ* cells is comparable to *mus81Δ*. Bars represent standard deviation from 3 independent experiments. Statistically significant differences as determined by a two-tailed Student’s t-test are indicated.

Broken replication forks are predominantly repaired by sister chromatid recombination **(**SCR), in which the resected seDSB at the broken fork invades the intact sister chromatid to restart DNA synthesis [42–44]. Mre11-Rad50-Nbs1 (MRN) protein complex and Ctp1/CtIP initiate resection, whilst strand invasion requires Rad51, Rad52, and Rad54, along with the Rad55-Rad57 or Swi5/Sfr1 recombination mediators. The functional profiling screen identified Ctp1 as important for chromium resistance. Fitness assays confirmed that *mre11Δ* and *rad51Δ* cells are also highly sensitive to chromium (Fig 5A). Rad51 functions less efficiently in the absence of the Rad55/Rad57 complex [45], and as predicted, *rad57Δ* cells were sensitive to chromium (Fig 5A).

SCR repair of seDSBs at collapsed forks results in sister chromatid exchange (SCE), whereas HR repair of ‘two-ended’ DSBs occurs by gene conversion via synthesis-dependent strand annealing (SDSA), which does not involve SCE. Both pathways require DSB resection and strand invasion into the sister chromatid, but only SCR depends on resolution of a DNA joint molecule, either a D-loop or Holliday junction (HJ), by Mus81-Eme1 resolvase [42, 46–48]. This difference explains why *mus81Δ* cells are quite sensitive to agents such as CPT that collapse replication forks but are completely insensitive to clastogens that break both DNA strands, such as ionizing radiation [49–51]. As predicted by our data, *mus81Δ* cells were quite sensitive to chromium-6 (Fig 5A), confirming that it creates DNA lesions that are repaired by SCR.

Rqh1 is a RecQ family DNA helicase, homologous to human WRN/BLM helicases, which is critical for replication stress survival is essential in the absence of Mus81 [50, 51]. Consistent with our other data, we found that *rqh1Δ* cells are chromate-sensitive (Fig 5B).

### Cds1 checkpoint response suppresses Rad52 foci formation in chromium-treated cells

**Fig 6:**
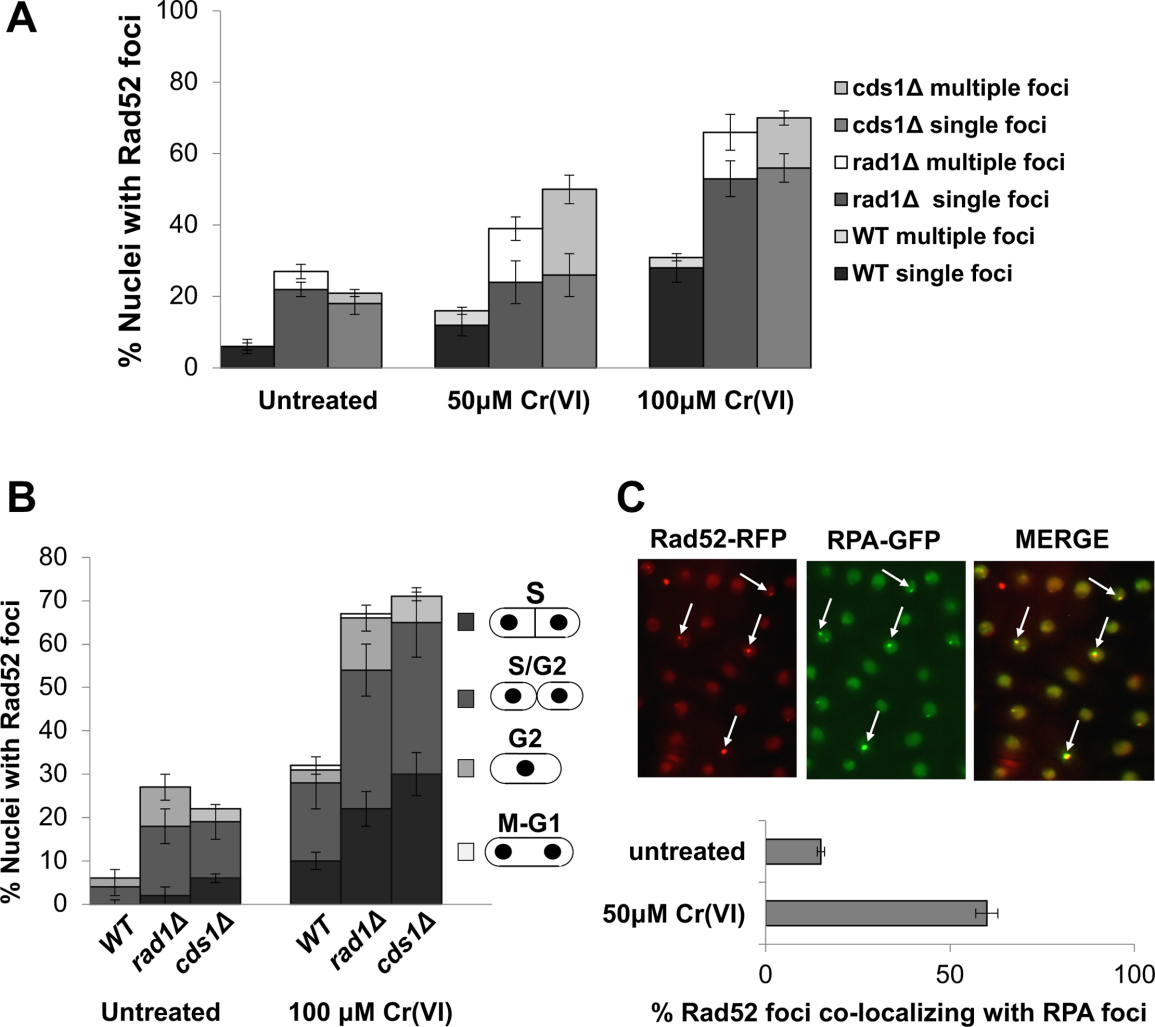
Increased Rad52 foci in Cr(VI)-treated *cds1Δ* and *rad1Δ* cells. **(A)** In comparison to untreated cells, there is an ~ 3-fold increase in Rad52-YFP nuclear foci in WT cells treated with chromium. Cells defective for intra S-checkpoint signaling and stabilization of replication fork show a 2-fold increase in Rad52 foci formation even in the absence genotoxins. The *cds1Δ* and *rad1Δ* mutants showed Rad52 foci formation in 70% of the cells, as compared to 30% in WT. **(B)** Quantification according to cell cycle stage indicated maximum Rad52 foci in S and early G2 phase cells in wild type, *cds1Δ* and *rad1Δ* mutants. **(C)** Co-localization of Rad11-GFP and Rad52-RFP foci on treatment with Cr(VI) indicates the spatial and temporal overlap of replication stress events with recombination. Arrows indicate co-localization. All foci were scored in live cells at 25°C after treatment with 100 μM Cr(VI) in EMM for 3h. Cells were washed with fresh media before scoring. Error bars are indicative of standard deviation from three independent experiments.

During HR repair, Rad52 binds ssDNA to displace RPA and assist formation of Rad51 nucleofilaments [46–48]. We monitored Rad52 nuclear foci to further investigate the source of the DSBs and the requirement for HR repair in Cr(VI)-treated cells [52, 53]. For these studies, we used cells that expressed Rad52 tagged with yellow fluorescent protein (YFP) expressed from the endogenous locus [52]. Chromium treated caused a large increase in cells with Rad52-YFP nuclear (Fig 6A). The large majority of these cells were in S or early G2 phase (Fig 6B). These data support the model that replication fork collapse is the primary source of DSBs in Cr(VI)-treated cells.

To further investigate the roles of checkpoint responses in Cr(VI)-treated cells, we monitored Rad52-YFP foci in *cds1Δ* and *rad1Δ* mutants (Fig 6A). Both mutants had more Rad52 foci in untreated conditions, but Cr(VI) treatment caused a further large increase of Rad52 foci, with the large majority appearing during S-phase or early G2 phase (Fig 6B). These data suggest that the Rad1 and Cds1-dependent replication checkpoint response helps to prevent replication fork collapse in Cr(VI)-treated cells.

To explore the relationship between the RPA and Rad52 foci in Cr(VI)-treated cells, we analyzed a strain that expressed the RPA-GFP and Rad52 tagged with red fluorescent protein (RFP) [54]. Prior to Cr(VI) exposure, only ~17% of the Rad52-RFP foci overlapped with RPA-GFP foci. This value increased to ~60% after a 3-hour exposure to 100 μM K_2_Cr_2_O_7_ (Fig 6C). These data suggest that that Cr(VI) causes replication fork collapse, leading to formation of resected seDSBs that are bound by RPA and Rad52.

### NER-independent activity of Rad16-Swi10 (XPF-ERRC1) endonuclease contributes to chromium resistance

**Fig 7:**
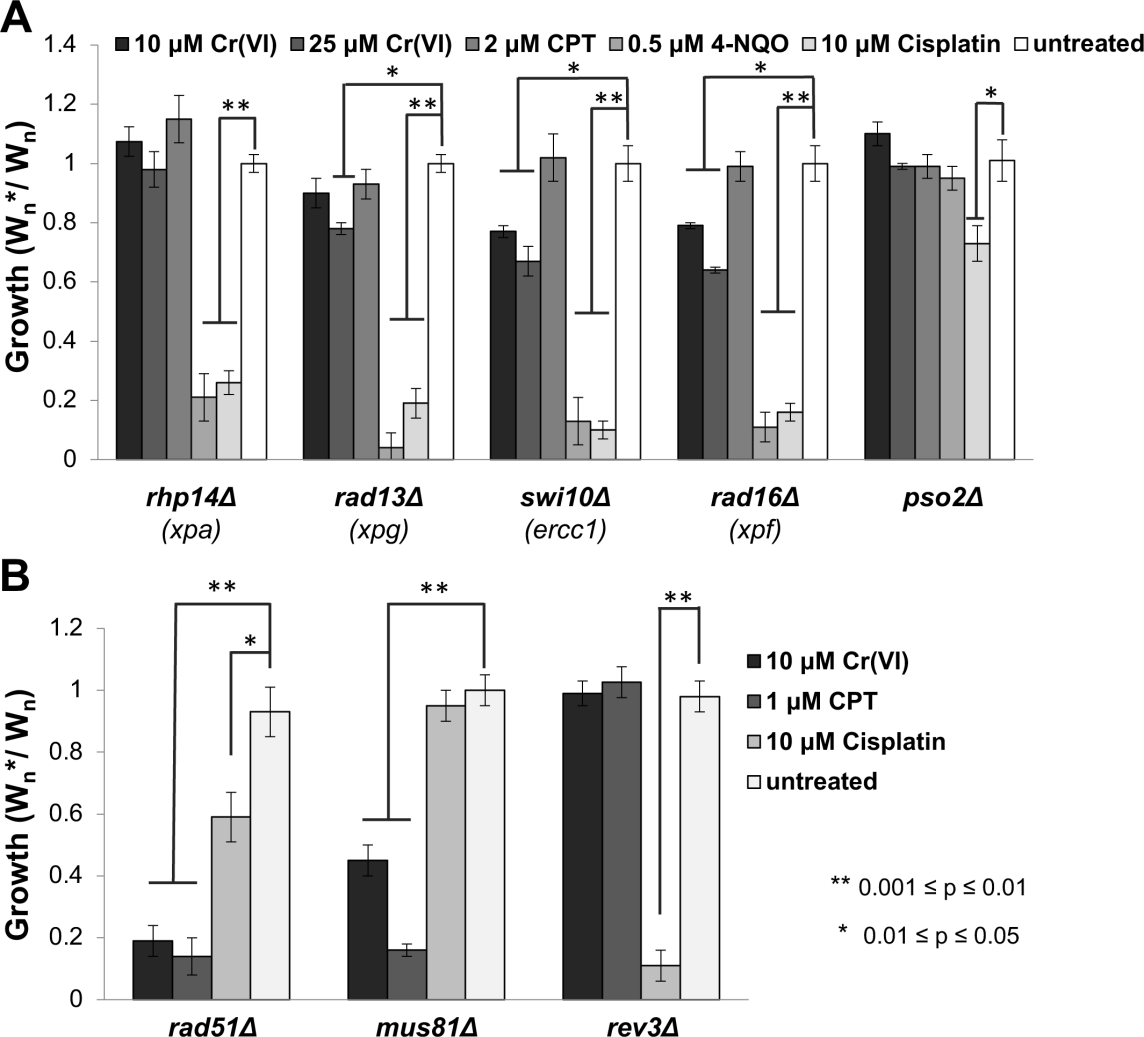
NER-independent activity of Rad16-Swi10 endonuclease contributes to chromium resistance. (A) Competitive growth assays were performed with the indicated concentrations of chromate, the UV mimetic 4-NQO, the DNA crosslinker cisplatin, and CPT. Cells lacking the NER proteins Rhp14/XPA, Rad13/XPG, Swi10/ERCC1 or Rad16/XPF are all highly sensitive to 4-NQO and cisplatin, but insensitive to CPT. Only cells lacking the Swi10 or Rad16 subunits of the 3’ flap endonuclease are clearly sensitive to chromate, whereas those lacking the Rad13 3’ incision endonuclease appear to have weak sensitivity to chromate. Cells lacking Rhp14 are insensitive to chromate. The graph also shows that Pso2 endonuclease is required for resistance to cisplatin but not chromate, CPT or 4-NQO. (B) Cells lacking Rev3 TLS DNA polymerase zeta are sensitive to 10μM cisplatin, but not 10μM chromate or 1μM CPT. Cells lacking Mus81 or Rad51 are sensitive to CPT and chromate resistance, but only cells lacking Rad51 are sensitive to cisplatin. Bars indicate standard deviation from 3 independent experiments. Statistically significant differences as determined by a two-tailed Student’s t-test are indicated.

Nucleotide excision repair (NER) was reported to be important for removal of Cr(III)-DNA adducts or survival of chromium exposure in mammalian and avian cells [55–57], yet NER mutants were not identified in our screen. To investigate this difference we first analyzed XPA (Rhp14 in fission yeast), which is a key NER protein that detects distortions in the DNA backbone and thus is crucial for DNA damage recognition and assembly of the pre-incision complex [58]. Rhp14/XPA was confirmed as critical for survival of cisplatin and 4-nitroquinoline (4-NQO), which is a UV-mimetic, yet *rhp14Δ* cells were completely insensitive to chromium (Fig 7A). We also analyzed XPG (Rad13 in fission yeast), which is the NER endonuclease that cleaves 3’ to DNA lesions [59]. In this case we detected only mild sensitivity to 25μM Cr(VI), which contrasts with the extreme sensitivity to cisplatin and 4-NQO (Fig 7A). Next, we investigated XPF-ERCC1 (Rad16-Swi10 in fission yeast), which incises 5’ to DNA lesion during NER [59]. Rad16-Swi10 was also crucial for cisplatin and 4-NQO resistance, as expected, but unlike the other NER proteins it also proved to be important for chromium resistance, with the mutants displaying significant sensitivity to 10μM Cr(VI) (Fig 7A). Collectively, these data indicate that an NER-independent activity of Rad16-Swi10 endonuclease plays an important role in repair of Cr-induced DNA lesions.

### Post-replication repair mutants are insensitive to chromium

Rad16-Swi10 (XPF-ERCC1) endonuclease has an NER-independent function required for replication-coupled repair of ICLs [60]. However, ICLs did not appear to be an important factor in chromium toxicity, as mutants lacking Rhp14 (XPA) or Rad13 (XPG) were acutely sensitive to the ICL-causing agent cisplatin but were insensitive or only weakly sensitive to chromium (Fig 7A). To explore this question further, we examined the role of the translesion DNA synthesis (TLS) DNA polymerase zeta (Rev3), which is critical for ICL repair [61–63]. We found that Rev3 is essential for cisplatin resistance but fully dispensable for chromium-6 resistance (Fig 7B).

**Fig 8:**
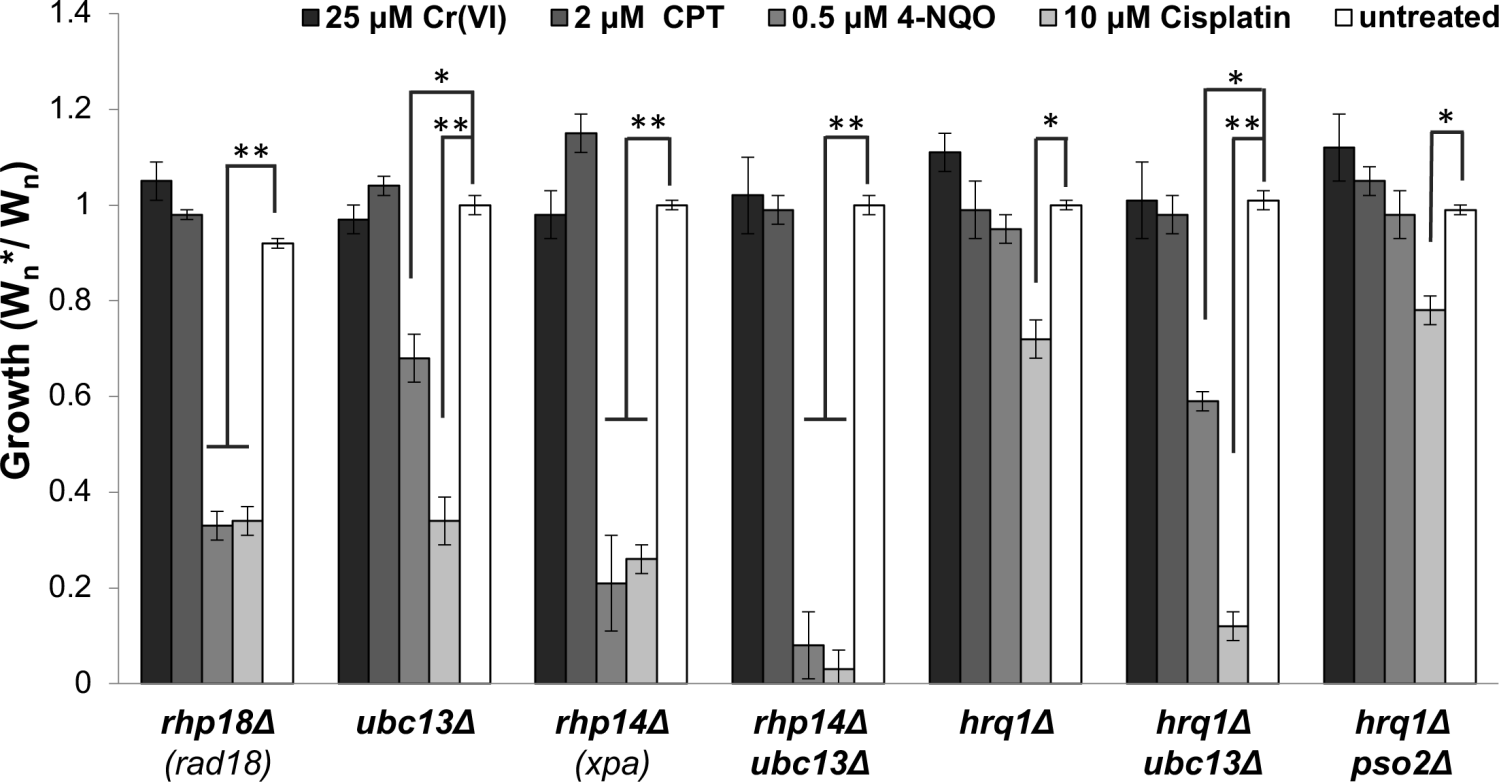
Post-replication repair mutants are insensitive to chromium. Competitive growth assays were performed with the indicated concentrations of chromate, CPT, 4-NQO, and cisplatin. Cells lacking the PRR proteins Rhp18/RAD18 or Ubc13 are sensitive to 4-NQO and cisplatin but not chromate or CPT. Elimination or Rhp14/XPA further increases 4-NQO and cisplatin sensitivities in cells lacking Ubc13. Cells lacking Hrq1/RECQL4 DNA helicase are only sensitive to cisplatin, whilst even co-elimination of Hrq1 and Ubc13 fails to cause any chromate or CPT sensitivity. Bars indicate standard deviation from 3 independent experiments. Statistically significant differences as determined by a two-tailed Student’s t-test are indicated.

Post replication repair (PRR) mechanisms permit replication of DNA having replisome-blocking DNA lesions. There are two major mechanisms of PRR: translesion DNA synthesis, which uses TLS polymerases to replicate across the DNA lesion, and template switching (TS), which uses the sister chromatid as a replication template to bypass the DNA lesion. In *S*. *cerevisiae*, both pathways depend on the monoubiquitylation of proliferating cell nuclear antigen (PCNA) catalyzed by the Rad6-Rad18 E2/E3 ubiquitin ligase complex, whereas only TS furthers depends on PCNA polyubiquitination catalyzed by Ubc13-Mms2-Rad5 E2/E3 ubiquitin ligase [64, 65]. However, in *S*. *pombe* both pathways are defective in the absence of PCNA polyubiquitination [66]. We observed that an *rhp18Δ* mutant, which lacks the fission yeast ortholog of Rad18, was acutely sensitive to cisplatin and 4-NQO, but insensitive to CPT and chromium-6 (Fig 8). Similarly, a *ubc13Δ* mutant was moderately sensitive to 4-NQO and highly sensitive to cisplatin, but insensitive to CPT and hexavalent chromium (Fig 8). These findings show that the PRR pathways for DNA damage tolerance are not required for chromium resistance.

NER and PRR are distinct, non-overlapping mechanisms of coping with bulky intrastrand crosslinks. To investigate whether this genetic redundancy was obscuring roles for these pathways in mediating chromium-6 resistance, we examined the phenotypes of *rhp14Δ ubc13Δ* double mutants. As predicted, this strain was substantially more sensitive to 4-NQO and cisplatin than either corresponding single mutant (Fig 8). However, *rhp14Δ ubc13Δ* cells remained fully resistant to chromium-6 toxicity.

Fission yeast Hrq1, an ortholog of human RECQL4 and a member of the RecQ family DNA helicases, was shown to support nucleotide excision repair of DNA damage caused by cisplatin and, in certain genetic contexts, UV light [67]. Accordingly, we observed that *hrq1Δ* cells were moderately sensitive to cisplatin; moreover, loss of Hrq1 enhanced cisplatin sensitivity in an *ubc13Δ* background (Fig 8). In contrast, the *hrq1Δ ubc13Δ* strain was completely resistant to toxicity caused by 25μM Cr(VI).

Taken together, these data establish that the DNA lesions induced by chromium, which cause replication stress and fork collapse, are otherwise efficiently repaired or tolerated in the absence of PRR and NER.

### A Tdp1-Pnk1 pathway repairs chromium-DNA lesions

**Fig 9:**
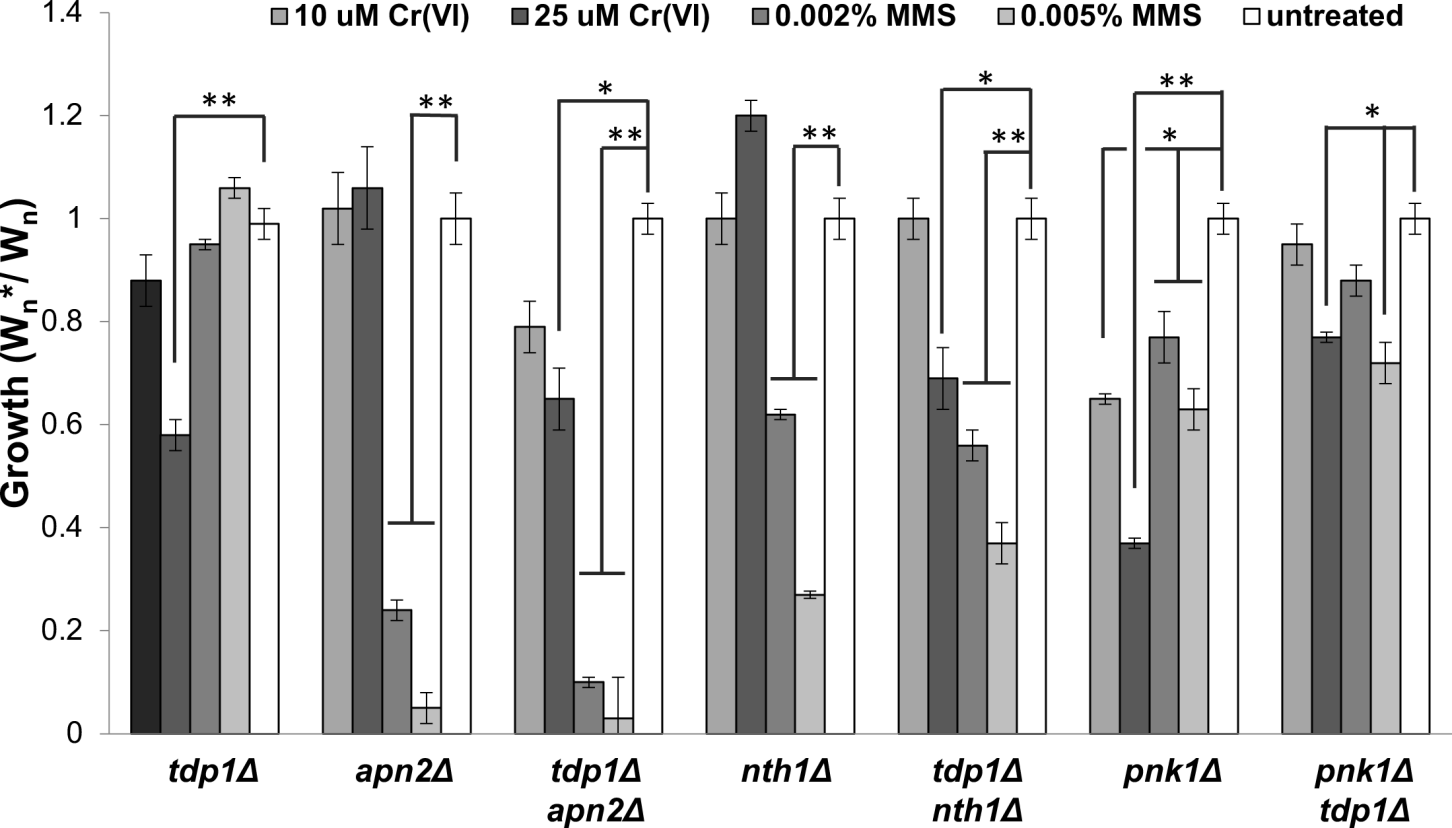
Tdp1 and Pnk1 repair Cr-induced DNA lesions. Competitive growth assays were performed with the indicated concentrations of chromate or MMS. Cells lacking Tdp1 are only sensitive to chromate, whereas cells lacking the BER proteins Apn2 or Nth1 are only sensitive to MMS. There appear to be no genetic interactions of *tdp1Δ* with *apn2Δ* or *nth1Δ*; for example, the chromate sensitivity of the *tdp1Δ apn2Δ* strain is similar to *tdp1Δ*. Cells lacking Pnk1 are sensitive to both chromate and MMS, but these sensitivities are partially rescued by *tdp1Δ*. Bars indicate standard deviation from 3 independent experiments. Statistically significant differences as determined by a two-tailed Student’s t-test are indicated.

DNA lesions produced by chromium are thought to include oxidized bases and apurinic/apyrimidinic (AP) sites, which are primarily fixed by base excision repair (BER) [10, 11, 68]. However, it is unclear if BER plays a significant role in chromium resistance. The major BER pathway requires cleavage of AP sites by an AP-lyase to yield 3′-deoxyribose phosphate (3′-dRP) termini, which are then processed by an AP-endonuclease to produce 3′-OH termini. In fission yeast lab strains these activities are provided by Nth1 and Apn2, respectively [69–71]. We confirmed that *nth1Δ* and *apn2Δ* cells are highly sensitive to MMS, but we found they are completely insensitive to chromium (Fig 9). Thus, BER is not required for chromium resistance in fission yeast.

Tdp1 is a tyrosyl-DNA phosphodiesterase that hydrolyzes the phosphodiester bond between a DNA 3′ end and a tyrosyl moiety [72, 73]. Tdp1 cleaves this bond in topoisomerase I-DNA covalent complexes (Top1cc); indeed, fission yeast *tdp1Δ* mutants display significant sensitivity to CPT that is fully suppressed by eliminating Top1 [74]. Tdp1 has not been implicated in chromium resistance, and the *tdp1Δ* mutant was not identified as chromate-sensitive in the deletome screen performed with 10μM chromate (S1 Table), but we observed that *tdp1Δ* cells displayed significant sensitivity to 25μM Cr(VI) (Fig 9). Tdp1 also has a weak 3′-dRP processing activity, hence loss of Tdp1 further enhances the MMS sensitivity in *apn2Δ* cells [75, 76]. We confirmed this genetic interaction in fitness assay, but found that loss of Apn2 did not enhance the chromium sensitivity of *tdp1Δ* cells (Fig 9). The same relationships were observed in an *tdp1Δ nth1Δ* double mutant (Fig 9).

Tdp1 removal of Top1 covalent complexes (Top1cc) leaves a ssDNA nick with a 5’ hydroxyl and 3’ phosphate. Pnk1 polynucleotide 5’-kinase 3’-phosphatase (PNKP) processes these DNA ends to 5’-phosphate and 3’- hydroxyl, which are required for DNA ligation [76–78]. The Pnk1 3’-phosphatase activity is important for CPT resistance in fission yeast [76]. Consistent with the *tdp1Δ* data, we found that *pnk1Δ* cells ranked about 100^th^ most sensitive in the deletome screen performed with 10μM chromate (S1 Table). The flow cytometry–based growth competition assay confirmed that *pnk1Δ* cells are sensitive to chromium (Fig 9). In fact, *pnk1Δ* cells were considerably more sensitive to chromium than *tdp1Δ* cells, and *tdp1Δ pnk1Δ* cells were equivalent to *tdp1Δ* cells (Fig 9). The latter result suggests that Tdp1 is principally responsible for generating Pnk1 substrates from chromium-induced DNA lesions.

### Tdp1 and Rad16-Swi10 provide alternative pathways for repairing Top1-independent Cr-induced DNA lesions

**Fig 10:**
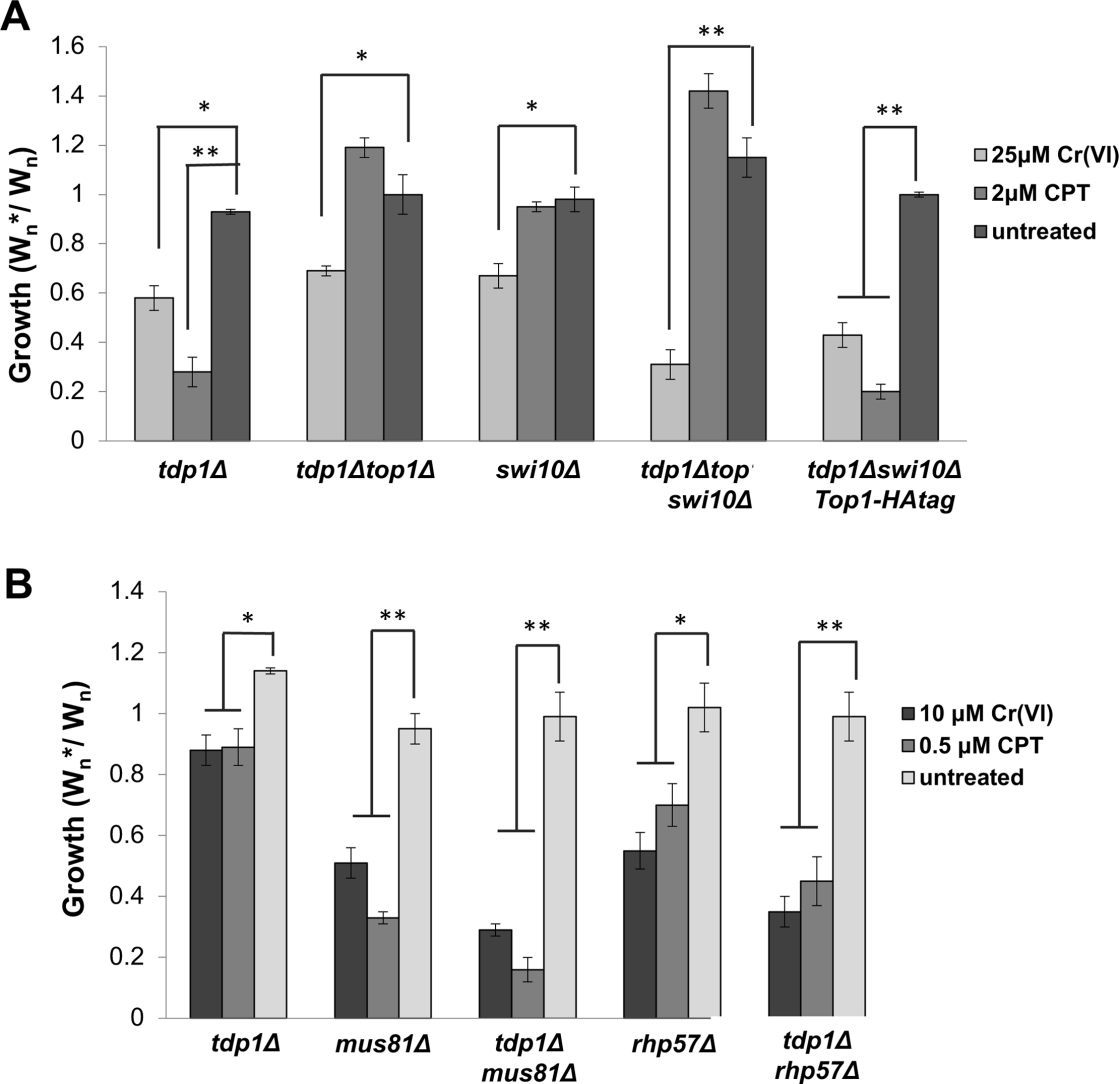
Tdp1 and Rad16-Swi10 provide alternative pathways for repairing Top1-independent Cr-induced DNA lesions. Competitive growth assays were performed with the indicated concentrations of chromate or CPT. (A) Elimination of Top1 suppresses *tdp1Δ* CPT sensitivity but not chromate sensitivity. Elimination of Top1, which suppresses *tdp1Δ swi10Δ* synthetic lethality, suppresses CPT sensitivity but not chromate sensitivity. Partial inactivation of Top1 caused by attachment of an HA epitope tag (*top1-HA)* suppresses the *tdp1Δ swi10Δ* synthetic lethality but these cells remain acutely sensitive to chromate. (B) Loss of Tdp1 enhances chromate and CPT sensitivities in *mu81Δ* and *rhp57Δ* backgrounds. Bars indicate standard deviation from 3 independent experiments. Statistically significant differences as determined by a two-tailed Student’s t-test are indicated.

In *S*. *cerevisiae* and *S*. *pombe*, the 3’-flap endonuclease activity of Rad16-Swi10 provides a backup pathway for repairing Top1cc lesions in the absence of Tdp1 [79, 80]. In fact, the synthetic lethal interaction of *tdp1Δ* and *swi10Δ* mutations is suppressed by the *top1Δ* mutation [79]. Our flow cytometry-based assay confirmed that the *top1Δ* mutation suppressed the CPT sensitivity of a *tdp1Δ* mutant (Fig 10A). However, the *top1Δ* mutation failed to suppress the chromium sensitivity of the *tdp1Δ* mutant (Fig 10A). As predicted, the *tdp1Δ top1Δ swi10Δ* mutant was insensitive to CPT. However, it was acutely sensitive to chromium, and more so than either *tdp1Δ top1Δ* or *swi10Δ* strains. To confirm these results, we analyzed a *tdp1Δ swi10Δ top1-HA* strain, in which the partial inactivation of Top1 caused by the C-terminal HA tag is sufficient to suppress the *tdp1Δ swi10Δ* synthetic lethality [79], yet there is sufficient Top1 activity to cause CPT sensitivity (Fig 10A). We observed that *tdp1Δ swi10Δ top1-HA* strain was highly sensitive to chromium-6, again exceeding the sensitivity of *tdp1Δ top1Δ* or *swi10Δ* strains (Fig 10A). From these results, we conclude that Tdp1 and Rad16-Swi10 likely initiate independent pathways eliminating Cr-DNA adducts, analogous to their roles in eliminating Top1cc.

These data suggested that SSB repair via the Tdp1-Pnk1 pathway plays an important role in chromium resistance. We hypothesized that in the absence of Tdp1, the SSBs are more likely to persist into S-phase, leading to replication fork collapse. According to this model, defects in Tdp1 and SCR should synergistically enhance chromium sensitivity. In support of this model, we found that *tdp1Δ mus81Δ* and *tdp1Δ rhp57Δ* mutants were more sensitive to chromium than the corresponding single mutants (Fig 10B).

## Discussion

In this study, we used the unbiased approach of deletome functional profiling to uncover the genetic requirements for preventing Cr(VI) toxicity in fission yeast. This screen identified a select group of 68 genes comprising about 2% of the tested mutants. Strikingly, hierarchical cluster analysis of GI scores revealed a toxicogenomic profile that closely resembles genotoxins that cause replication stress and fork collapse. Indeed, GO term enrichment analysis identified Rad3/ATR-dependent checkpoints as being crucial for Cr(VI) resistance. Competitive growth assays confirmed the importance of these genes and extended the list to include essentially all the genes that play key roles in the DNA replication stress and DNA damage checkpoints. The requirements for Cds1/CHK2 and Chk1, which are activated by Rad3, were supported by data showing that both kinases are phosphorylated in Cr(VI)-treated cells. Cds1 activation is restricted to S-phase because its mediator Mrc1 is only expressed in this phase of the cell cycle [81]. Our studies revealed that chromium-induced activation of Chk1 also occurs in S-phase. Thus, checkpoints that detect stalled and collapsed replication forks are crucial for surviving chromium exposure in fission yeast.

Collapsed forks are efficiently repaired by sister chromatid recombination, and our assays confirmed that key SCR genes are crucial for Cr(VI) resistance. These genes encode subunits of the MRN-Ctp1 complex that initiates DSB resection, Rad51 recombinase and its mediators, and Mus81-Eme1 resolvase. The requirement for Mus81 is especially informative because in mitotic cells it is specifically required to complete the repair of seDSBs at broken replication forks [42]. Thus, the most straightforward interpretation of our data is that Cr(VI)-induced DSBs occur principally during S-phase through replication fork collapse. This conclusion is supported by the cell cycle analyses of RPA/Rad52 foci formation and Chk1 phosphorylation.

The most recent studies of mammalian cells indicated that chromium specifically elicits an ATR-dependent checkpoint response, whereas reports of ATM activation were ascribed to an ascorbate deficiency in the culture media, resulting in excessive oxidative stress [21]. As ATM/Tel1 binds the MRN complex at DSBs [82, 83], these data indicated that Cr-DNA lesions do not result in the formation of frank DSBs that are bound and processed by the MRN complex and CtIP/Ctp1 [21]. This conclusion would also be consistent with studies of chicken DT40 cells reporting that CtIP is not required for chromate resistance [57]. However, our data establish that MRN and Ctp1 are essential for chromate resistance in fission yeast, which is consistent with MRN-Ctp1 being critical for repair of a broken replication fork [42]. Thus, our data more closely align with mammalian cell studies indicating that the Mre11 complex is important for responding Cr-induced DNA lesions in [84].

Chromium creates interstrand crosslinks (ICLs) in vitro but there is uncertainty as to whether ICLs are physiologically relevant chromium-induced DNA lesions in mammalian cells [55, 85]. Cisplatin, which creates intrastrand and interstrand crosslinks, was acutely lethal in mutants lacking Rhp14/XPA or Rev3, or lacking both Rhp14 and Ubc13, and yet these strains displayed no sensitivity to chromium. These data strongly indicate that chromate does not lead to substantial ICL formation in fission yeast.

Chromate creates DNA SSBs in vitro and there are increased SSBs in DNA extracted from mammalian cells grown in the presence of chromate [6, 9, 12]. However, current evidence indicates that most of these breaks are produced by excision repair or during extraction of Cr-adducted DNA in alkaline conditions [86]. Cells lines that lack XRCC1, which is a molecular scaffold involved in SSB repair (SSBR), are moderately sensitive to chromate. However, this sensitivity is suppressed by increasing ascorbate concentrations, suggesting that an unnatural ascorbate deficiency increases SSB formation by chromate [87]. In fission yeast, the Tdp1-dependent pathway of SSBR is fully active in the absence of an XRCC1 ortholog. This pathway repairs SSBs with 3’-blocked DNA ends, most notably those blocked by Top1CC in CPT treated cells. We found that Tdp1 is required for resistance to chromate toxicity. Consistent with observation, we found that Pnk1, which functions directly downstream of Tdp1 to restore 3’-hydroxyl ends to SSBs, is also important for chromate resistance. In fact, *pnk1Δ* cells are more sensitive to chromate than are *tdp1Δ* cells, and the *tdp1Δ* genotype is epistatic in the *tdp1Δ pnk1ΔΔ* double mutant. These data indicate two things: (1) the 3’-phophate SSBs generated by Tdp1 are not easily repaired in the absence of Pnk1, (2) there is an alternative pathway for repairing the chromate-induced SSBs that partially compensates for a Tdp1 deficiency. In the case or Top1cc’s, this pathway requires Rad16-Swi10 (XPF-ERCC1) in an NER-independent role. Strikingly, Rad16-Swi10 has a NER-independent function in chromate resistance, and elimination of this activity enhances chromate sensitivity in an *tdp1Δ* cells. Importantly, this experiment was performed in a *top1Δ* background because spontaneous Top1CCs are lethal in cells lacking Tdp1 and Rad16-Swi10. Thus, the toxic chromate-induced SSB processed by Tdp1 and Rad16-Swi10 does not involve Top1.

Even in the presence of fully functional Tdp1 and Rad16-Swi10-dependent pathways of repairing CPT-induced Top1cc lesions, some of these SSBs persist into S-phase, during which they cause replication fork collapse. This inefficiency in SSB repair during interphase likely explains the critical requirement for HR proteins such as Mre11 and Mus81 in CPT-treated cells [51, 88]. In the absence of Tdp1, more Top1cc’s are likely to remain unrepaired as cells enter S-phase, which is consistent with enhanced requirement for HR proteins and Mus81 in *tdp1Δ* cells treated with CPT [79]. We observed a parallel set of relationships in cells treated with chromate, most notably, *tdp1Δ mus81Δ* cells were substantially more sensitive to chromate than either single mutant. These data suggest that unrepaired SSBs with blocked 3’ ends are responsible for a substantial fraction of the collapsed forks and seDSBs in chromate-treated cells.

Tdp1 is thought to have weak AP-lyase and AP-endonuclease activities that are able to cleave AP and 3’-dRP sites, respectively [89]. However, in terms of MMS sensitivity, a role for Tdp1 is only observed in the absence of Apn2 AP-endonuclease, which is critical for repair of AP sites [75, 76]. We found that that *nth1Δ* and *apn2Δ* cells are highly sensitive to MMS but completely insensitive to chromium, indicating that the weak AP-lyase and AP-endonuclease activities of Tdp1 are unlikely to be important for chromium resistance.

It is interesting that GO term enrichment analysis of the 387 genes identified in the CrO_3_ sensitivity screens of the *S*. *cerevisiae* deletome did not highlight DDRs as being critical for Cr(VI) resistance, but instead uncovered important roles for vacuolar function and vesicle fusion (S2 and S3 Tables) [28, 29]. These data suggest that Cr(VI) compound sequestration or efflux mediated by vacuoles might be crucial in budding yeast. Another possibility is that conversion of CrO_3_ into chromic acid significantly acidified the growth media. Indeed, the GO term “vacuolar acidification” was strongly enriched amongst the mutants that were sensitive to 900 μM CrO_3_ [29]. DDR-related genes were identified in the more recent CrO_3_ screen of the *S*. *cerevisiae* deletome [28], including 5 members of the RAD52 epistasis group, but GO terms associated with DSB repair were not significantly enriched after applying the Bonferroni correction to eliminate false positives [90, 91]. We speculate that the failure to significantly enrich DDR-related GO terms in the *S*. *cerevisiae* Cr(VI) screens reflects idiosyncrasies of the screening methods, as the DNA lesions formed in Cr(VI)-treated *S*. *cerevisiae* and *S*. *pombe* are likely similar overall. However, there could be also be some important differences in the DNA lesion spectrum resulting from divergent mechanisms of chromate metabolism. Other differences in cell physiology, cell cycle parameters or genetic redundancies might also play a role. In future studies it will be important to determine why, for example, NHEJ appears to be important for chromate resistance in *S*. *cerevisiae* [92] but not in *S*. *pombe* (S1 Table).

**Fig 11:**
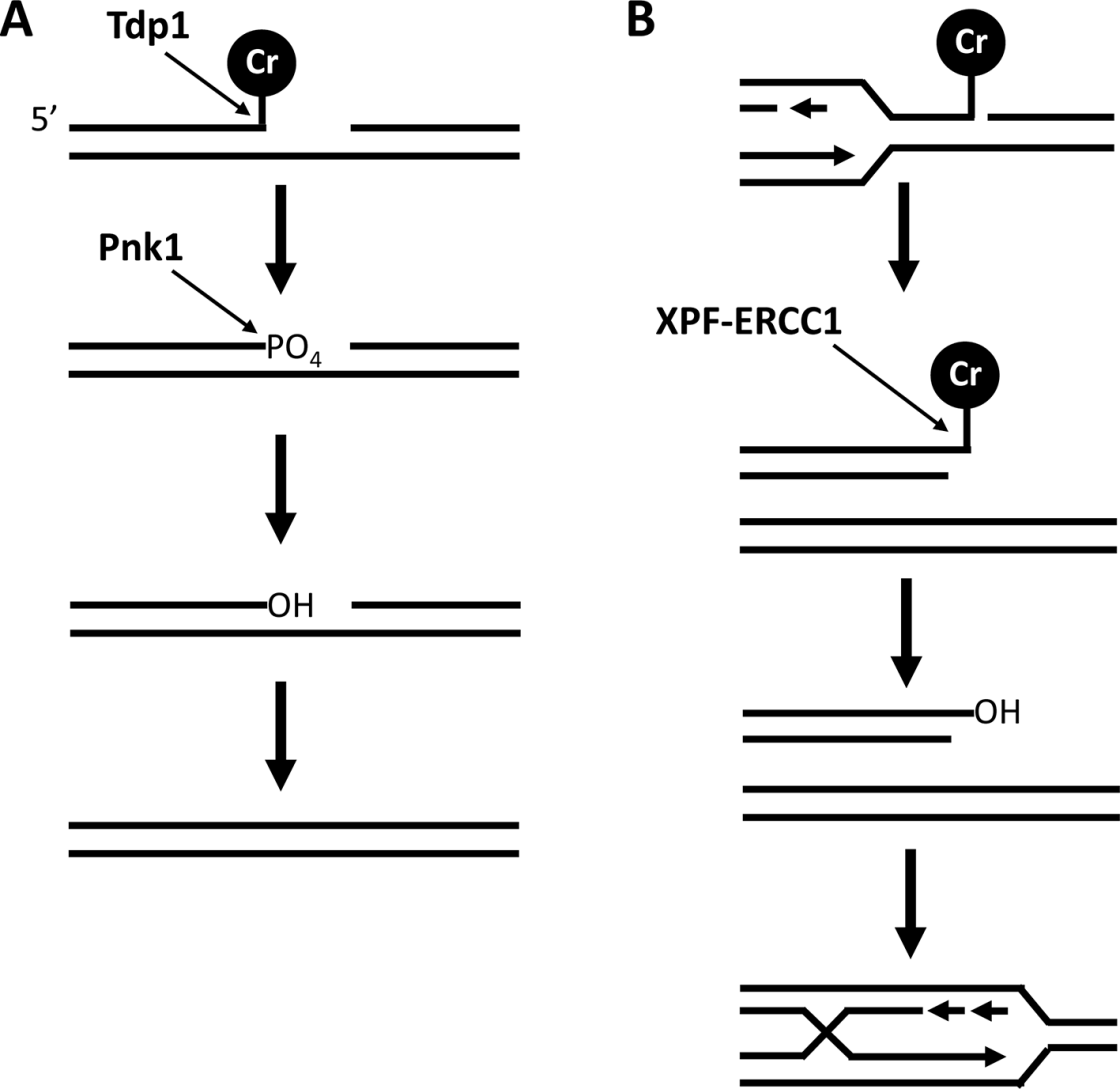
Model for repair of Cr-DNA lesions by Tdp1-Pnk1 and Rad16-Swi10 (XPF-ERCC1) pathways. (A) Tdp1 processes Cr-DNA SSBs to generate SSBs with a 3’-PO_4_ terminus that is a substrate for Pnk1. (B) Alternatively, these Cr-DNA lesions may be processed by Rad16-Swi10 (XPF-ERCC1). In the model shown, this cleavage occurs at an seDSB that is formed by replication fork collapse. After removal of Cr-DNA lesion, the seDSB is repaired by SCR requiring key HR proteins such as MRN-Ctp1 and Rad51, followed by resolution of the Holliday junction or D-loop by Mus81-Eme1 endonuclease.

In summary, our data shows that an important fraction of Cr-induced DNA lesions are repaired by Tdp1-Pnk1 or an NER-independent activity of Rad16-Swi10 (XPF-ERCC1) endonuclease (Fig 11). Given the well-documented ability of these enzymes to repair 3’-blocked SSBs, we propose that Top1-independent 3’-blocked SSBs are substantially responsible for chromate toxicity. A subset of these SSBs and possibly other Cr-induced DNA lesions are apparently inefficiently repaired during interphase, which accounts for the crucial requirements for Rad3/ATR-dependent checkpoints that detect stalled and collapse replication forks, and the Mus81-dependent SCR pathway of repairing collapsed forks. We propose that these events underlie chromium genotoxicity and likely contribute to the carcinogenic properties of hexavalent chromium.

## Materials and methods

### Strains and genetic methods

The *S*. *pombe* haploid deletion library from Bioneer has genes deleted with the KanMX4 cassette in the genetic background *h*^+^ *leu1-32 ura4-D18 ade6-M210/M216* [25]. Standard *S*. *pombe* genetic procedures and media were used as described previously [93, 94]. Cells were grown in YES (yeast extract with glucose and supplements) media or EMM2 (Edinburgh minimal media), at 30°C unless described otherwise. The strain MBY2440 (*swo1-GFP*:*ura4*^+^ *leu1-32 ura4-D18 h*^*-*^*)* was a kind gift from Prof. Mohan Balasubramanian [95]. It was modified to generate strain AG5495 (*swo1-GFP*:*NatMX6 leu1-32 ura4-D18* h^+^) that was used for our flow cytometry-based competitive growth assay. Briefly, the 0.795 kb *ura4* ORF [96] was replaced with NatMX6 antibiotic marker using pFA6a-NatMX6 as template and the primers Ura4-Forward (5’-TGTCGTGTTT-TCTTACCGTA-TTGTCCTACC-AAGAACCTCTT-TTTTGCTTGG-ATCGAAATTAA-AGGTTTAAAA-GCAAAGTTCG-GATCCCCGGG-TTAATTAA-3’) and Ura4-Reverse (5’-AAAGAGTACT-TGTATACTAA-TTCTAAATGC-CTTCTGACAT-AAAACGCCTA-GGAAAACAAA-CGCAAACAAG-GCATCGACTT-GAATTCGAGC-TCGTTTAAAC-3’). The PCR product was transformed into MBY2440 by electroporation; putative NatMX6 colonies were selected and verified with PCR as well as lack of growth on EMM2 (Edinburgh minimal media) plates supplemented with leucine, adenine and histidine (LAH). All other strains used for the flow cytometry-based competitive growth assay were also in the *h*^*+*^ *leu1-32 ura4-D18* background unless otherwise noted. All strains used for *S*. *pombe* genetic and biochemical studies are listed in S4 Table.

### Screening and confirmation of chromium-sensitive mutants

Detailed procedures for deletion library pool construction, deep sequencing and barcode data analysis were as described [26, 27]. Briefly, frozen aliquots of the pooled deletion strains were recovered in YES media and allowed to grow for 5 hours at 30°C. Cultures were split and adjusted to 10 μM K_2_Cr_2_O_7_ or left untreated. Cultures were grown for ~5 generations before harvesting by centrifugation. Samples were lysed in TE buffer (10 mM Tris-HCl, 1 mM EDTA, pH 8.0), genomic DNA was extracted and the barcodes were amplified. PCR products were purified and mixed in equal molar amounts. Standard single-end sequencing primers were used for 42 cycles of sequencing in an Illumina Genome Analyzer II. Barcode sequencing data have been deposited at NCBI Sequence Read Archive (http://www.ncbi.nlm.nih.gov/sra/) under the accession number SRP108456.

A Growth Inhibition (GI) score was calculated for each mutant in the library to determine its toxin sensitivity [27]. Briefly, a normalized log2 ratio of barcode sequencing counts in control vs. treatment sample was calculated for each mutant. This calculation normalizes values for slow growing mutants. The ratio was divided by the number of cell doublings (five for the screens analyzed here) to obtain the GI score. For chromium-sensitive mutants, we expect GI scores higher than 0 because of the depletion of these mutants from the deletion pool under the treatment condition. The mutants that completely failed to grow under the treatment condition are expected to have GI scores around 1. To select treatment-sensitive mutants, a robust Z-score was calculated for each gene, which is the deviation of its GI score from the median GI score expressed in the number of the normalized interquartile range (NIQR). We performed 2 independent screens with chromium. The first screen was performed with Version 1.0 of the Bioneer haploid collection. The second screen was performed with an updated library consisting of 3004 mutants. Consequently a few hundred genes only have a GI score for only one screen.

A tail area-based false discovery rate (FDR) cutoff of 0.1 [97] was used to identify mutants sensitive to chromate. Fifty-two (52) genes were identified that passed the FDR cutoff in both screens for chromium sensitivity. Mutants that met the FDR cutoff in only one screen underwent spot assay analysis. Mutants from YES agar plates supplemented with 150 mg/l G418 were incubated in YES liquid media in a 96-well plate and grown at 30°C for 2 or 3 days to reach saturation density. Cultures were then diluted 20-fold (OD_600_ ~0.4) and incubated for another 3 hours. Ten-fold serial dilutions of the cell cultures were spotted onto YES agar with or without K_2_Cr_2_O_7_. Chromium mutant sensitivity was assessed on plates containing 10, 50, 100 or 200 μM K_2_Cr_2_O_7_. Note that chromium appears to be more toxic at lower concentrations in liquid media compared to agar media, thus the K_2_Cr_2_O_7_ concentrations used for the spot dilution assays were empirically determined. Metal sensitivity was assessed by visual inspection. This analysis identified an additional 17 Cr(VI)-sensitive mutants. For these mutants, expressivity (high, medium or low) was based on the lowest concentration of chromate to which the mutant was clearly sensitive. Note that these expressivity categories are not directly comparable to those determined using GI scores. Eight of the 51 mutants found to be sensitive in both pooled library screens appeared chromate-insensitive in YES agar. This discrepancy might reflect an actual difference in liquid versus agar media.

### GO term enrichment and identification of homologous proteins

GO term enrichment analysis was performed using the web-based tool AnGeLi [31] available at http://bahlerweb.cs.ucl.ac.uk/cgi-bin/GLA/GLA_input, or the generic GO term finder available from the Bioinformatics Group at the Lewis-Sigler Institute, Princeton University (http://go.princeton.edu). Cluster 3.0 was used for hierarchical clustering analysis and Java TreeView was used for visualization of the cluster analysis. Pair-wise average-linkage and uncentered correlation was chosen for analysis. On-line software YOGY (http://www.bahlerlab.info/YOGY/) [98] was used for retrieving homologous proteins from human and *S*. *cerevisiae*.

### Competitive growth assays by flow cytometry

We adapted methods first established for *S*. *cerevisiae* [32, 33]. Overnight cultures of wild type (WT) cells expressing GFP-tagged Swo1 (WT-GFP^+^) or not expressing GFP (WT-GFP^-^) and individual mutant strains that were not expressing GFP (M-GFP^-^) were diluted to OD_600_ = 0.2 and allowed to grow to OD_600_ of 0.8–1.2 (~1 x 10^7^ cells/ml) in YES media at 30°C. Approximately equal numbers of cells (based on individual OD_600_ values) from GFP^+^ and GFP^-^ strains were mixed together in a 1:1 ratio and re-inoculated into 5 ml of fresh YES media with or without genotoxins at a starting OD_600_ of 0.01 [33]. For these studies, we used 5–25 μM K_2_Cr_2_O_7_ as indicated in the figures, 5 μM CdSO_4_, or the indicated concentrations of genotoxins, which were well tolerated by wild type and had minimal impacts on GFP fluorescence and cellular autofluorescence (S1 Fig). Cells were grown to an OD_600_ of ~2.0 (~7 generations). Samples of 1 ml (~1 x 10^7^ cells) were centrifuged at 3,000 rpm for 3 minutes, washed and resuspended in 1 ml of Phosphate Buffered Saline (1X PBS buffer) and stored at 4°C for further analysis. These cells were stained with DAPI (4',6-diamidino-2-phenylindole) at a final concentration of 2.5 μg/ml and incubated for 10 minutes at room temperature before analyzing using a FACS Calibur flow cytometer (BD Biosciences) with an air-cooled 488 nm argon ion laser [99]. The 450/50 Pacific Blue filter was used to gate out cells that were unable to exclude DAPI from the cytoplasm [99]. Samples were analyzed using 530/30 (FITC) and 585/42 (PE) filters. Non-cellular debris and clumped cells were excluded using Forward Scatter (FSC) and Side Scatter (SSC) gates. As some mutants become elongated in the presence of genotoxins, a FSC-A versus FSC-H correlation was better suited for doublets discrimination. Typically, a 3–5% reduction was observed in the total population after these filters. WT (GFP^+^) cells exhibiting loss of GFP fluorescence after these filters was found to be ~ 0.1% in the absence of toxicant metals, ~ 0.3–0.5% in 5–10 μM K_2_Cr_2_O_7_, and < 1% for 5 μM CdSO_4_. GFP^+^ and GFP^-^ cells were readily separable due to low spectral overlap between FITC and PE. Rare events that appeared as GFP^+^/GFP^-^ (FITC intensity between 10^2^−10^3^ arbitrary units) were gated out during the analysis. Data represented here are an average of three experimental replicates.

The following values were determined by flow cytometry: mutant fraction in the absence (W_m_) or presence (W_m_*) of toxicant = M-GFP^-^/ M-GFP^-^ + WT-GFP^+^; wild type fraction in the absence (W_r_) or presence (W_r_*) of toxicant = WT-GFP^-^/ WT-GFP^-^ + WT-GFP^+^. To correct for the small percentage of WT-GFP^+^ that lost GFP fluorescence we normalized values in the absence (W_n_ = W_m_ / W_r_) or presence (W_n_* = W_m_* / W_r_*) of toxicant. Finally, growth ratio in the presence of toxicant = W_n_* / W_n_.

Wild type GFP^-^ cells were <1% in any given situation, thus variability in mutant cell count due to these would be rare (S1 Fig). However, to account for this variability, instead of using absolute cell count as a measure of mutant growth, normalized growth for mutants (W_N_) was calculated from the ratio of mutant cells count (W_M_) vs wild type cell count (W_R_) over the course of competition W_N_ = W_M_/W_R_. WT-untagged vs WT-tagged indicated that WT-GFP^+^ had an absolute cell count of 42.5%. Since autofluorescence was detected at < 1%, the lowest detectable normalized growth for mutants acceptable for our assay was set at W_N_ = 0.03.

Growth ratio for mutants was calculated as a ratio of their normalized or relative growth in the presence of toxicant vs without toxicant (W_n_* / W_N_).

### Microscopy

Rad52-YFP, Rad52-RFP and Rad11-GFP (aka RPA-GFP) were expressed from their endogenous loci. All strains were grown in EMM2 at 25°C to mid-log phase for foci quantification. For genotoxin treatments cells were grown in the presence of 50 or 100 μM K_2_Cr_2_O_7_ or 30 μM CPT in DMSO for 2 hours at 25°C, then washed and resuspended in fresh EMM2 before foci quantification. At least 200 nuclei were scored in three independent experiments. All foci analysis was conducted on live (unfixed) cells. Cells were photographed using Nikon Eclipse E800 microscope equipped with a Lumenera Infiniti 3-1M camera. ImageJ software was used for all analysis.

### Cell cycle block and release

Temperature sensitive *cdc25–22* cells were grown to early log phase (OD600 = ~0.4) at 25°C in YES media. The temperature was increased to 35°C for 3 hours to arrest cells in G2 phase, after which K_2_Cr_2_O_7_ was added to a final concentration of 100 μM and incubated for an additional 2 hours before cells were released at 25°C to synchronously re-enter the cell cycle. 50 ml samples were harvested every 30 minutes for 2 hours. Cell pellets collected by centrifugation were rapidly frozen at -80°C to later probe for Chk1 phosphorylation. One sample was left at 35°C and harvested at the last time point (120 minutes). For septation index measurements, parallel samples of cells were fixed in 1% formaldehyde, washed in water and stained with fluorescein.

### Immunoblot analysis

For Chk1 and Cds1 phosphorylation analysis, exponentially growing cells in YES media were treated with 200 μM K_2_Cr_2_O_7_, NaAsO_2_ or CdSO_4_ for 2 or 4 hours at 30°C. Cells were also treated with 20 mM HU or 30 μM CPT for positive controls. Whole-cell extracts from 50 ml cultures were resuspended in standard lysis buffer (50 mM Tris pH 8.0, 150 mM NaCl, 2.5 mM EDTA, 0.002% NP-40, 50mM NaF, 1X Roche cOmplete protease inhibitor cocktail) and disrupted with glass beads in an MP Biomedicals FastPrep-24 instrument using 4 bursts of 20 seconds at a speed setting of 5.0. Samples containing 120–150 mg of protein were resolved on 10% SDS-PAGE with acrylamide/bisacrylamide ratio of 99:1. Proteins were transferred to nitrocellulose membranes, blocked with 5% milk in TBS buffer with 0.05% Tween and probed with an anti-HA (12CA5) antibody (Roche). Anti-α-Tubulin from Sigma (T5168) was used as loading control.

## Supporting information

S1 Fig Flow cytometry-based competitive growth assay. **(A)** GFP fluorescence signals observed in *WT swo1-GFP* cells grown in the absence or presence of toxicant. **(B)** Wild type GFP+ and GFP- cells are readily scored by flow cytometry in the absence or presence of toxicant. In these experiments GFP+ cells displayed a modest growth advantage over their GFP- counterpart. **(C)** Wild type (GFP+) cells that lost the GFP fluorescence signal varied between 0.1–1.0%. Thus, variability in mutant count due to this factor was negligible.
(TIF)

S1 Table Chromate-sensitive mutants. ***Sheet 1*** lists GI scores from 2 functional profiling experiments (Column C and D). Average rank (column E) is based on the “1026” experiment data. Spot assay (column F) indicates the lowest K_2_Cr_2_O_7_ concentration (10, 50, 100 or 200 μM) in which the mutant displayed sensitivity relative to wild type. Expressivity (column G) indicates whether the mutants displayed high, medium or low K_2_Cr_2_O_7_ sensitivity determined by functional profiling by Bar-seq (rows 2–52) or spot assay (rows 53–69). Column H describes the gene product for the identified mutants. Column I-K lists the corresponding homologs in *S*. *cerevisiae* (59 out of 68) and humans (52 out of 68). Column L indicates 21 genes required for CPT, HU or UV resistance identified using the same functional profiling platform (see Sheet 2) [27]. Column M indicates the genes required for arsenic or cadmium tolerance (10 out of 68 genes) identified using the same functional profiling platform [26]. Column N indicates genes whose budding yeast orthologs were identified in previous *S*. *cerevisiae* chromium-6 functional profiling screens (see Sheet 3) [28, 29]. ***Sheet 2*** lists chromate-sensitive mutants that were also scored as sensitive to other genotoxins (CPT, HU, or UV) or TBZ in a previous functional profiling screen [27]. ***Sheet 3*** compares S. pombe and S. cerevisiae Cr(VI) functional profiling screens. ***Sheet 4*** lists growth inhibition (GI) scores for functional profiling experiments performed with hexavalent chromium.
(XLS)

S2 Table GO Biological processes enriched for 214 S. cerevisiae Cr(VI)-sensitive mutants identified by Jin et al., 2008. (PDF)

S3 Table GO Biological processes enriched for 205 *S. cerevisiae* Cr(VI)-sensitive mutants identified by Johnson et al., 2016. (PDF)

S4 Table *S. pombe* strains used in this study. (XLSX)
