# Supplementary figures and images for: Tdp1 processes chromate-induced single-strand DNA breaks that collapse replication forks
Source: PLoS Genet. 2018 Aug 27;14(8):e1007595. doi: 10.1371/journal.pgen.1007595 (PMC6128646; doi:10.1371/journal.pgen.1007595)

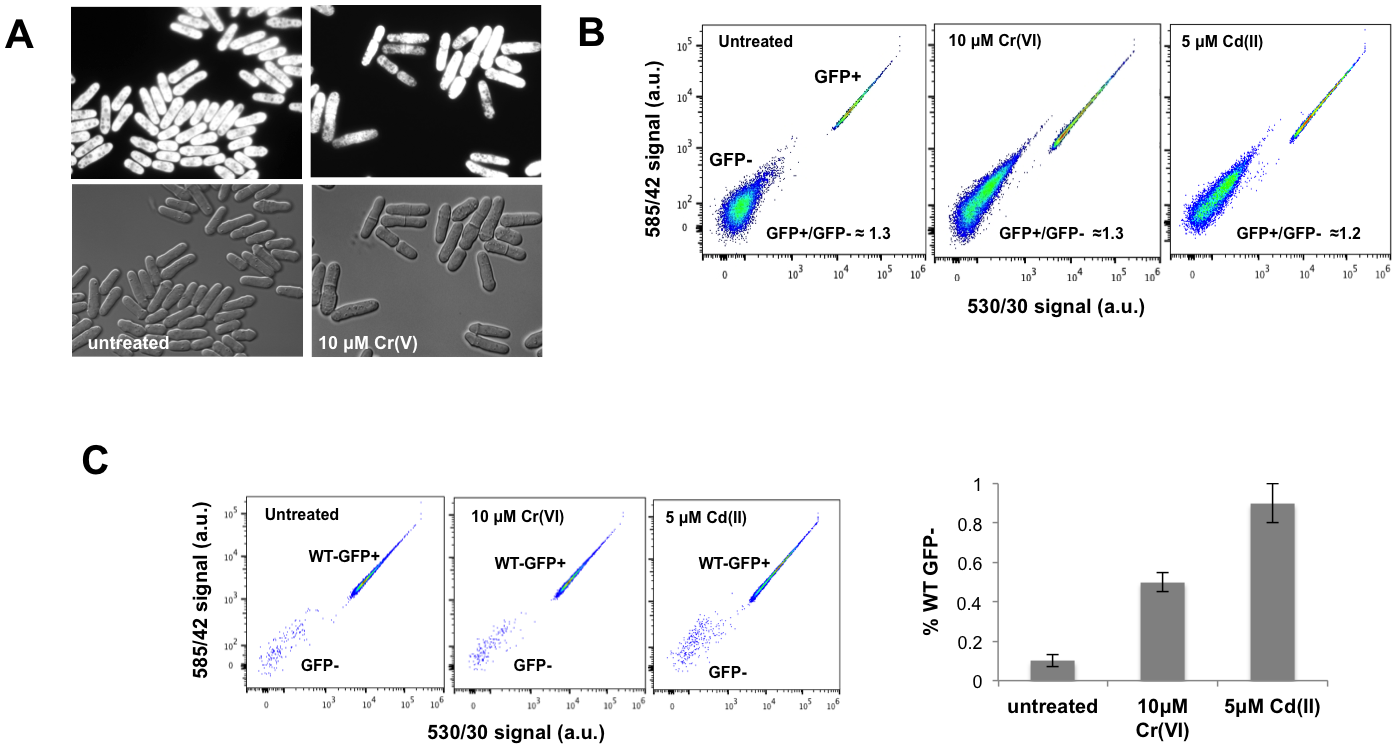

Supplement: S1 Fig — (A) GFP fluorescence signals observed in WT swo1-GFP cells grown in the absence or presence of toxicant. (B) Wild type GFP+ and GFP- cells are readily scored by flow cytometry in the absence or presence of toxicant. In these experiments GFP+ cells displayed a modest growth advantage over their GFP- counterpart. (C) Wild type (GFP+) cells that lost the GFP fluorescence signal varied between 0.1–1.0%. Thus, variability in mutant count due to this factor was negligible. (TIF) [file pgen.1007595.s001.tif]
