# Supplementary material for: Tdp1 processes chromate-induced single-strand DNA breaks that collapse replication forks
Source: PLoS Genet. 2018 Aug 27;14(8):e1007595. doi: 10.1371/journal.pgen.1007595 (PMC6128646; doi:10.1371/journal.pgen.1007595)
Supplement: S2 Table — (PDF) [file pgen.1007595.s003.pdf]

Table S2. GO Biological Processes enriched for 214 *S. cerevisiae* Cr(VI)-sensitive mutants identified by Jin et al., 2008.

Most Enriched GO Biological Processes	Corrected p-value	Cluster Frequency (214 genes)	Genome Frequency (4879 genes)	Genes Annotated
Vacuolar acidification	5.46e-09	13 (13.7%)	179 (3.7%)	VPS53, DRS2, VPS29, STP22, COG8, VPS41, DID2, PEP3, PEP12, DID4, ATG11, VPS36, VPS8, VPS54, SNF7, SNF8, VPS1, VPS16, VMA3, RAS2, VPS33, VTC1, VTA1, ELO2, VPS28, APL5, VPS4, VPS25
Vesicle fusion	4.89e-06	14 (6.8%)	48 (1.0%)	DID2, DID4, ELO2, PEP3, SNF7, SNF8, STP22, VPS16, VPS25, VPS28, VPS36, VPS4, VPS8, VTA1
tRNA aminoacylation	0.00170	8 (3.9%)	22 (0.5%)	PEP12, PEP3, SEC22, VAM6, VPS16, VPS33, VPS41, VPS8
Vacuolar transport	0.00018	25 (11.7%)	179 (3.7%)	PEP12, PEP3, SEC22, VAM6, VPS16, VPS33, VPS41, VPS8

GO term enrichment performed against the S. cerevisiae homozygous deletion collection 4879 genes, with Bonferroni correction, P-value cutoff = 0.01, FDR = 0% for all data in table, 0 false positives.
